# Ox-LDL induces a non-inflammatory response enriched for coronary artery disease risk in human endothelial cells

**DOI:** 10.1038/s41598-025-07763-3

**Published:** 2025-07-01

**Authors:** Jiahao Jiang, Thomas K. Hiron, Anil Chalisey, Yashaswat Malhotra, Thomas Agbaedeng, Chris A. O’Callaghan

**Affiliations:** 1 Centre for Human Genetics, Nuffield Department of Medicine, University of Oxford, Oxford, UK; 2 Present Address: Cardiovascular Disease Initiative, The Broad Institute of MIT and Harvard, Cambridge, MA USA; 3 Present Address: Department of Physiology, Anatomy & Genetics, University of Oxford, Oxford, UK; 4 Present Address: Cambridge University Hospitals NHS Foundation Trust, Cambridge, UK

**Keywords:** Cardiovascular diseases, Mechanisms of disease, Epigenomics, Functional genomics

## Abstract

Oxidised low-density lipoprotein cholesterol (ox-LDL) is critical in the initiation and progression of atherosclerosis. While excessive atherogenic lipids in the arterial intima can trigger endothelial dysfunction in advanced lesions, the response of endothelial cells to ox-LDL in the early stages of atherogenesis remains unclear. Here, we conducted a comprehensive, genome-wide multi-omics characterisation of the cellular response to ox-LDL in primary human aortic endothelial cells (HAECs). Our results reveal that the exposure of HAECs to ox-LDL leads to pathogenic changes in metabolism, transcriptome and epigenome, but in the absence of a typical inflammatory endothelial phenotype. An integrative analysis implicates the role of AP-1, NFE-2 and CEBP transcription factors in regulating ox-LDL-induced transcription. We further demonstrate that ox-LDL activates endothelial cell migration through the epigenomic rewiring of transcription factor binding. Notably, these ox-LDL-induced dynamic binding sites are enriched for the genetic risk of coronary artery disease, enabling the discovery of the gene-environment interaction of rs62172376 and ox-LDL at the *CALCRL*/*TFPI* locus. Collectively, our findings provide an unbiased understanding of the transcriptional regulation in endothelial cells in response to ox-LDL, together with its interaction with the genetic element of coronary artery disease.

**Supplementary Information:**

The online version contains supplementary material available at 10.1038/s41598-025-07763-3.

## Introduction

 Atherosclerosis is a chronic, multicellular disease characterised by the formation of lipid-rich plaques within the arterial wall, and is the primary underlying cause of coronary artery disease (CAD). Endothelial cells (ECs) constitute the first barrier of the arterial vasculature, and are central to the initiation and the progression of atherosclerosis. In health, ECs tightly control vascular tone, prevent blood clotting and maintain redox balance^1^; in disease or inflammatory conditions, damage to ECs triggers the recruitment of immune cells to the sub-endothelial space, further exacerbating the inflammatory and necrotic microenvironment within the arterial wall^2–5^.

Cholesterol-carrying low-density lipoprotein (LDL) particles are retained and chemically modified (e.g., notably by oxidation) in the vessel wall at lesion-prone sites during the initiation of atherosclerosis^6,7^. Due to their direct exposure to circulating blood, ECs have long been believed to be the first cell type to experience the pathogenic effects of LDL and its derivatives. Specifically, exposure to oxidised LDL (ox-LDL) has been linked to the development of pro-inflammatory endothelial phenotypes, characterised by increased expression of cell adhesion molecules such as ICAM-1 and VCAM-1 ^8-12^. However, contrasting results showing no or context-dependent endothelial activation after ox-LDL exposure were also observed using the same models^13–17^. Most of these early studies on ox-LDL and ECs were conducted using low-throughput approaches, with a narrow focus on specific pathways or functions hypothesised to be relevant. Therefore, these results have often been inconclusive. We reasoned that a comprehensive, unbiased assessment of the endothelial response to ox-LDL in primary human cells is crucial for a more complete understanding of this pathophysiological process underlying atherosclerosis.

The development of high-throughput multi-omics sequencing approaches enables unbiased, genome-wide investigation of the endothelial response to atherogenic lipids. One of the bioactive components of ox-LDL, oxidised 1-palmitoyl-2-arachidonoyl-sn-glycero-3-phosphocholine (ox-PAPC) has been extensively characterised^18–20^. In primary human aortic endothelial cells (HAECs), ox-PAPC has been shown to alter the transcriptome^21^epigenome^22^and metabolome^23^. While serving as a convenient synthetic alternative to the plasma-derived ox-LDL, ox-PAPC represents only a fraction of the lipid peroxidation products found in ox-LDL, and its pathogenic similarity with ox-LDL has not been systematically evaluated. Given that oxidised lipids do not act in isolation during atherosclerosis, measuring the overall effect of ox-LDL instead of its bioactive components might better reflect its multifaceted atherogenic properties in vivo.

Decades of large-scale, genome-wide association studies have revealed a large heritable component of CAD, with more than 330 risk loci reaching genome-wide significance^24–26^. ECs are significantly associated with the genetic risk of CAD^27,28^and a few EC-acting risk variants have been prioritised through fine-mapping studies^29,30^. Nevertheless, it remains poorly understood which other variants regulate which genes in endothelial cells to modulate disease risk. In particular, it is unclear whether any of these risk genes are regulated in response to atherogenic lipids in endothelial cells, or if they encode proteins that could potentially interact with these lipids.

In the current study, we employ a genome-wide, multi-omics approach to characterise the response of primary human endothelial cells to ox-LDL. We find only limited overlaps in the metabolic and transcriptomic effect between ox-LDL and ox-PAPC; ox-LDL-induced gene expression better aligns with the in vivo gene expression signatures in ECs from human atherosclerotic lesions. Using a combination of computational and experimental approaches, we find that ox-LDL triggers endothelial cell migration through epigenomic rewiring of transcription factor binding. In addition, we show that the endothelial response to ox-LDL is associated with the genetic risk of CAD, and prioritise CAD risk variants that overlap dynamic transcription factor binding sites.

## Results

### Ox-LDL accumulation triggers metabolic shift in HAECs

**Fig. 1:**
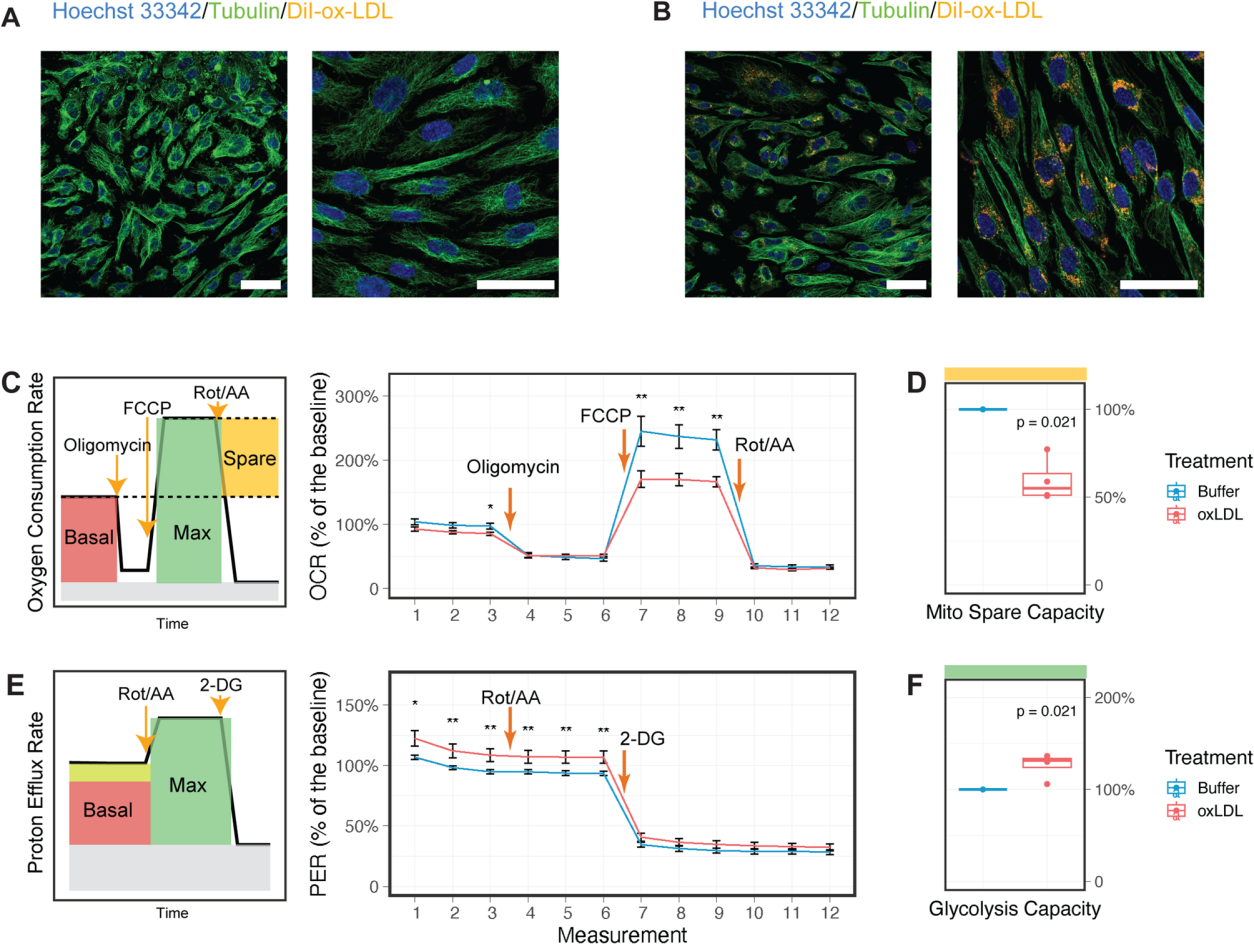
Exposure to ox-LDL induces metabolic stress in primary human aortic endothelial cells (HAECs). **A** and **B**, Representative images from immunofluorescence staining of unexposed (**A**) and ox-LDL-exposed (**B**) HAECs. Cells were treated with 50ug/mL of DiI-labelled ox-LDL or control buffer for 48 h before imaging. Scale bar: 50 μm. **C**, Oxygen consumption rate (OCR) of unexposed (blue) and ox-LDL-exposed (red) HAECs, shown as the mean value with the standard error for *n* = 4 biological replicates. Overview of the mitochondrial stress test assay is plotted to the left. **D**, The spare respiration capacity as marked in yellow in (**C**), normalised to the buffer group. **E**, Proton efflux rate (OCR) of unexposed (blue) and ox-LDL-exposed (red) HAECs, shown as the mean value with the standard error for *n* = 4 biological replicates. Overview of the glycolytic rate assay is plotted to the left. **F**, The glycolytic capacity as marked in green in (**E**), normalised to the buffer group. Seahorse assays were conducted with at least three technical repeats per biological replicate. P-values were calculated using Wilcoxon tests. * p-value < 0.05, ** p-value < 0.01.

It is well-recognized that endothelial cells play an important role in the transcytosis of lipids from the circulation to the sub-arterial space^31^. To test whether exposure to ox-LDL results in intracellular accumulation of lipids, primary human aortic endothelial cells (HAECs) were incubated with DiI-labelled ox-LDL and profiled using immunofluorescence microscopy. After 48 h of DiI-ox-LDL treatment, lipid droplets were clearly visible in the cytoplasm (Fig. 1A and B).

A previous study has reported that oxidised 1-palmitoyl-2-arachidonoyl-sn-glycero-3-phosphocholine (ox-PAPC), a synthesised component of ox-LDL, disrupts amino acid metabolism in endothelial cells^23^. To explore any potential metabolic consequences of ox-LDL exposure, we next measured the cellular respiratory rates and glycolytic rates using Seahorse assays (Fig. 1C and F).

While the basal respiration rate remains constant, ox-LDL treatment drastically reduced the spare respiration capacity the cells can utilize when the energy demand is high (Fig. 1C and D). In addition, cells exposed to ox-LDL displayed a significant increase in basal glycolytic rate (Fig. 1E and F); the addition of rotenone and antimycin A, which inhibits electron transport complexes and thereby shuts down mitochondrial respiration, did not further increase the glycolytic rate (Fig. 1E). These results contrast with the metabolic activation effect of ox-PAPC reported previously^23^suggesting that ox-LDL induces metabolic stress and pushes the energy balance toward glycolysis.

### Ox-LDL alone does not induce pro-inflammatory endothelial phenotypes

Previous studies have shown that ox-LDL induced endothelium dysfunction, an important pro-atherogenic process that is typically characterized by elevated expression of cellular adhesion molecules such as ICAM-1 and VCAM-1 ^9–12^. However, despite its intracellular accumulation and the metabolic consequences, ox-LDL-exposure was not found to affect the transcript nor protein level of ICAM-1 and VCAM-1 in HAECs (Figure S1A-D). Gene expression analysis of other inflammatory markers including *CCL2*, *TRAF2* and *IL6* also showed no sign of endothelial activation (Figure S1E). *CXCL8* was weakly up-regulated by ox-LDL but this was not significant after adjustment for multiple testing (FDR-adjusted *p* = 0.274, Figure S1E).

We noticed that in one of the previous reports^17^activation of inflammation was only observed when the lectin-type oxidized LDL receptor 1 (OLR1) was artificially overexpressed in HAECs. Despite being first identified as an endothelial receptor in bovine cells^33^*OLR1* is barely expressed in human endothelial cells from either disease-free vasculatures or atherosclerotic plaques (Figure S2A). Other receptors involved in lipid uptake, including *CD36*, *TLR4* and *LDLR*, were expressed at considerably higher levels (Figure S2B-D). Together, our results clearly demonstrate that OLR1-mediated endothelial activation is unlikely to be a direct consequence of ox-LDL exposure both in vitro and in vivo.

### Transcriptomic analysis reveals disrupted fatty-acid metabolism upon ox-LDL exposure

**Fig. 2:**
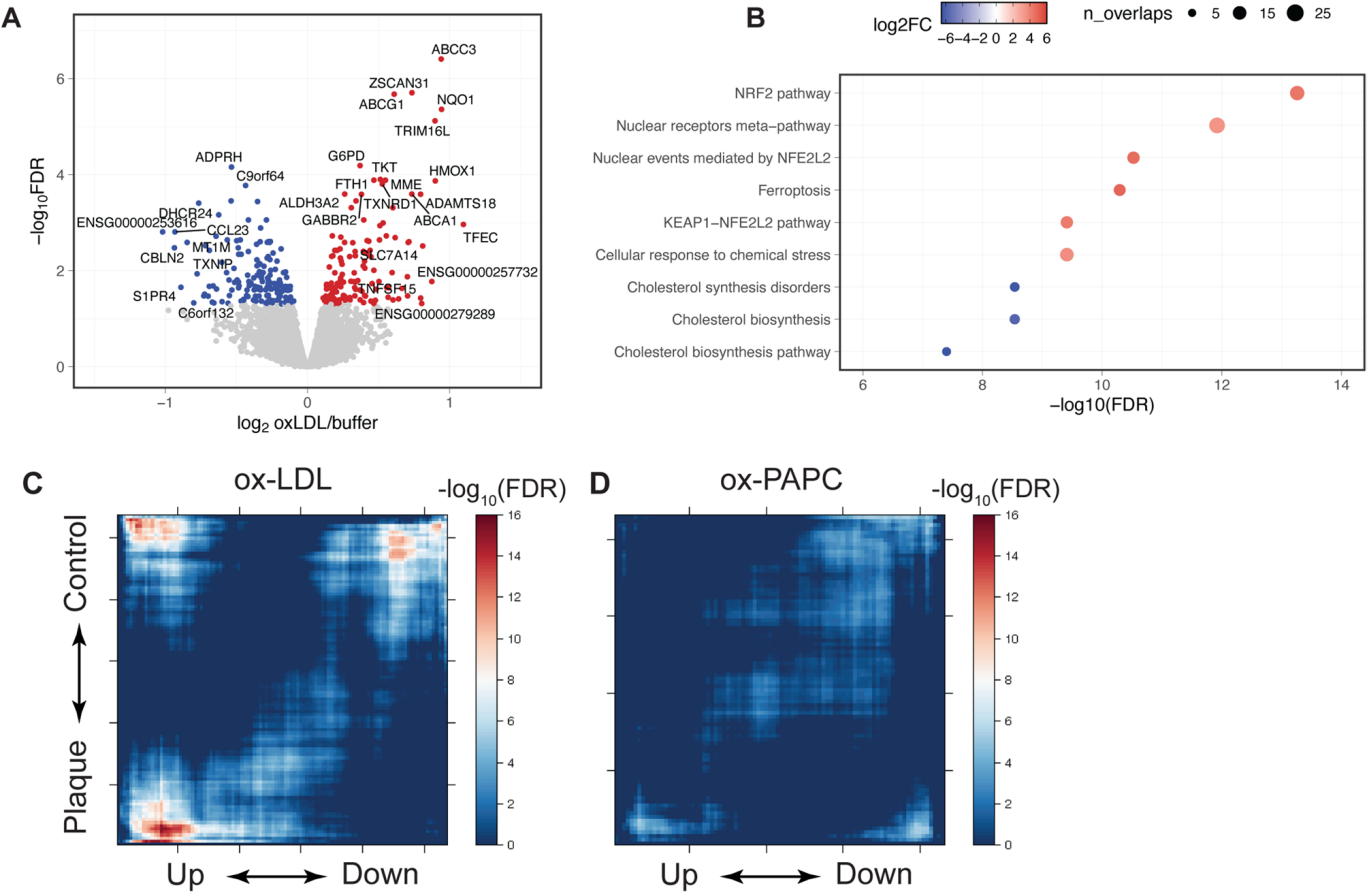
HAECs exposed to ox-LDL display transcriptomic signatures associated with endothelial cells in human atherosclerosis. A, Volcano plot showing differentially expressed genes up-regulated (red) and down-regulated (blue) by ox-LDL in HAECs. Genes with FDR > 0.05 are plotted in grey. B, MsigDB pathways enriched in the differentially expressed genes, ranked by statistical significance. Colour represents log2 fold change of pathway enrichment and size represents the number of overlapped genes in each pathway. C and D, Rank-rank hypergeometric overlap tests showing similarity between transcriptomic signatures related to plaque ECs (y axis), ox-LDL-treated HAECs (x axis in C) and ox-PAPC-treated HAECs (x axis in D). Note that genes were ranked by statistical significance of differential expression tests, so only the ends of x or y axis represent significant differentially-expressed genes. The colour denotes the significance of each hypergeometric test (-log_10_FDR), measuring the extent of overlap between the two gene set on a sliding basis.

To systematically characterize the impact of ox-LDL on gene expression in HAECs, we next performed RNA-seq analysis. After donor and batch effect correction, 304 genes were identified as differentially expressed under the FDR threshold of 0.05, with 144 genes being upregulated by ox-LDL and 160 genes downregulated (Fig. 2A). The most upregulated and most downregulated genes are listed in Tables 1 and 2. Among these genes were several with previously known roles in cholesterol efflux (e.g. *ABCA1*, *ABCC3*)^34^ and oxidative stress response (e.g. *NQO1*, *HMOX1*)^35^.

To investigate the biological pathways affected by ox-LDL, we performed an enrichment test for MSigDB and KEGG pathways. Again, we found no enrichment for pathways directly associated with endothelial inflammation (Fig. 2B and Figure S3A). Consistent with our observation in macrophages, ox-LDL treatment triggered the anti-oxidative-stress NFE2 signalling pathway and inhibited intrinsic cholesterol biosynthesis (Fig. 2B). One of the most up-regulated pathways following ox-LDL exposure was ferroptosis (Fig. 2B and Figure S3A), a form of regulated cell death that can be activated by the accumulation of oxidatively damaged lipid species^36^. Ferroptosis is recognised as a key trigger of endothelial dysfunction^37^and has been linked to increased atherosclerotic burden in mice^38^. Interestingly, our results demonstrate activation of ferroptosis in the absence of any pro-adhesive or activated endothelial phenotypes, which has not been previously reported in the literature.

The most upregulated protein-coding gene is *TFEC*, a member of the MiT/TFE transcription factor family. The MiT/TFE family has been linked to lysosome biogenesis in cancer cells^39^and we have recently demonstrated ox-LDL-induced expression of *MITF* in macrophages^32^. However, the cellular function of *TFEC* is poorly characterised, and no other MiT/TFE members were differentially expressed in ox-LDL-exposed endothelial cells.

**Table 1:** Top 10 most upregulated protein-coding genes by ox-LDL in HAECs.

Gene	log2$\:\left(\frac{\mathbf{o}\mathbf{x}\mathbf{L}\mathbf{D}\mathbf{L}}{\mathbf{B}\mathbf{u}\mathbf{f}\mathbf{f}\mathbf{e}\mathbf{r}}\right)$	FDR	Description
*TFEC*	1.097	1.09E-03	Transcription factor EC
*NQO1*	0.943	4.36E-06	NAD(P)H quinone dehydrogenase 1
*ABCC3*	0.940	3.91E-07	ATP binding cassette subfamily C member 3
*HMOX1*	0.899	1.35E-04	Heme oxygenase 1
*SLC7A14*	0.810	3.05E-03	Solute carrier family 7 member 14
*TNFSF15*	0.796	3.70E-02	TNF superfamily member 15
*ADAMTS18*	0.795	2.55E-04	ADAM Metallopeptidase With Thrombospondin Type 1 Motif 18
*ZSCAN31*	0.735	1.97E-06	Zinc finger and SCAN domain containing 31
*ABCA1*	0.734	2.53E-04	ATP binding cassette subfamily A member 1
*SLC7A11*	0.708	2.57E-03	Solute carrier family 7 member 11

**Table 2:** Top 10 most downregulated protein-coding genes by OxLDL in HAECs.

Gene	log2$\:\left(\frac{\mathbf{o}\mathbf{x}\mathbf{L}\mathbf{D}\mathbf{L}}{\mathbf{B}\mathbf{u}\mathbf{f}\mathbf{f}\mathbf{e}\mathbf{r}}\right)$	FDR	Description
*CBLN2*	−0.936	3.33E-03	Cerebellin 2 Precursor
*CCL23*	−0.932	1.55E-03	Chemokine ligand 23
*S1PR4*	−0.890	2.21E-02	Sphingosine-1-phosphate receptor 4
*MT1M*	−0.847	2.57E-03	Metallothionein 1E
*C6orf132*	−0.800	4.67E-02	Chromosome 6 Open Reading Frame 132
*TXNIP*	−0.776	1.16E-02	Thioredoxin-interacting protein
*DHCR24*	−0.765	3.91E-04	24-dehydrocholesterol reductase
*BDKRB2*	−0.729	3.34E-02	Bradykinin Receptor B2
*CD14*	−0.724	3.08E-02	CD14 molecule
*COL21A1*	−0.717	3.01E-03	Collagen Type XXI Alpha 1 Chain

### Exposure to ox-LDL but not ox-PAPC recapitulates the transcriptomic signatures associated with endothelial cells in human atherosclerosis

We next sought to confirm whether the transcriptomic changes induced by ox-LDL exposure ex vivo are present in human atherosclerosis in vivo. Briefly, the transcriptomic profiles of individual endothelial cells from human carotid plaques or adjacent disease-free vasculatures were analysed and aggregated into pseudo-bulk populations for differential expression tests, and the plaque expression signatures were compared with the ox-LDL expression signatures using a Rank-Rank Hypergeometric Overlap test^40^. The vast majority of genes expressed in vivo were also expressed in HAECs ex vivo, and levels of gene expression were strongly correlated between endothelial cells in vivo and ex vivo (Figure S3B).

It is important to note that the plaque endothelial cells were collected from advanced lesions, thus they were exposed to different microenvironments compared to our ox-LDL model. Despite this, we found that 65 out of the 304 differentially expressed genes induced by ox-LDL were also dysregulated in plaque (*p* = 3.25 × 10^−16^, Fisher’s Exact Test). Specifically, we identified significant overlaps at both ends of the differential expression gene list (Fig. 2C). This enrichment, however, was not evident in endothelial cells treated with ox-PAPC^22^ (Fig. 2D). Moreover, only limited overlaps were found between genes regulated by ox-LDL and those regulated by ox-PAPC (Figure S3C-D). Clearly, our ox-LDL-exposure model provides new information that better characterises the role of endothelial cells in human atherosclerosis.

### Integrative analysis highlights a shared ox-LDL-response network between endothelial cells and macrophages

We next sought to characterise the potential regulators that drive the endothelial response to ox-LDL. To map the potential cis-regulatory elements in HAECs, we performed high-throughput sequencing experiments with Assay for Transposase Accessible Chromatin (ATAC-seq) and chromatin immunoprecipitation (ChIP-seq) targeting the active enhancer marker H3K27 acetylation (H3K27ac). Surprisingly, although the chromatin accessibility at transcription start sites positively correlates with the gene expression (Figure S4A), no region was confidently identified as differentially accessible in the ox-LDL group compared to the control group (Figure S4B).

**Fig. 3:**
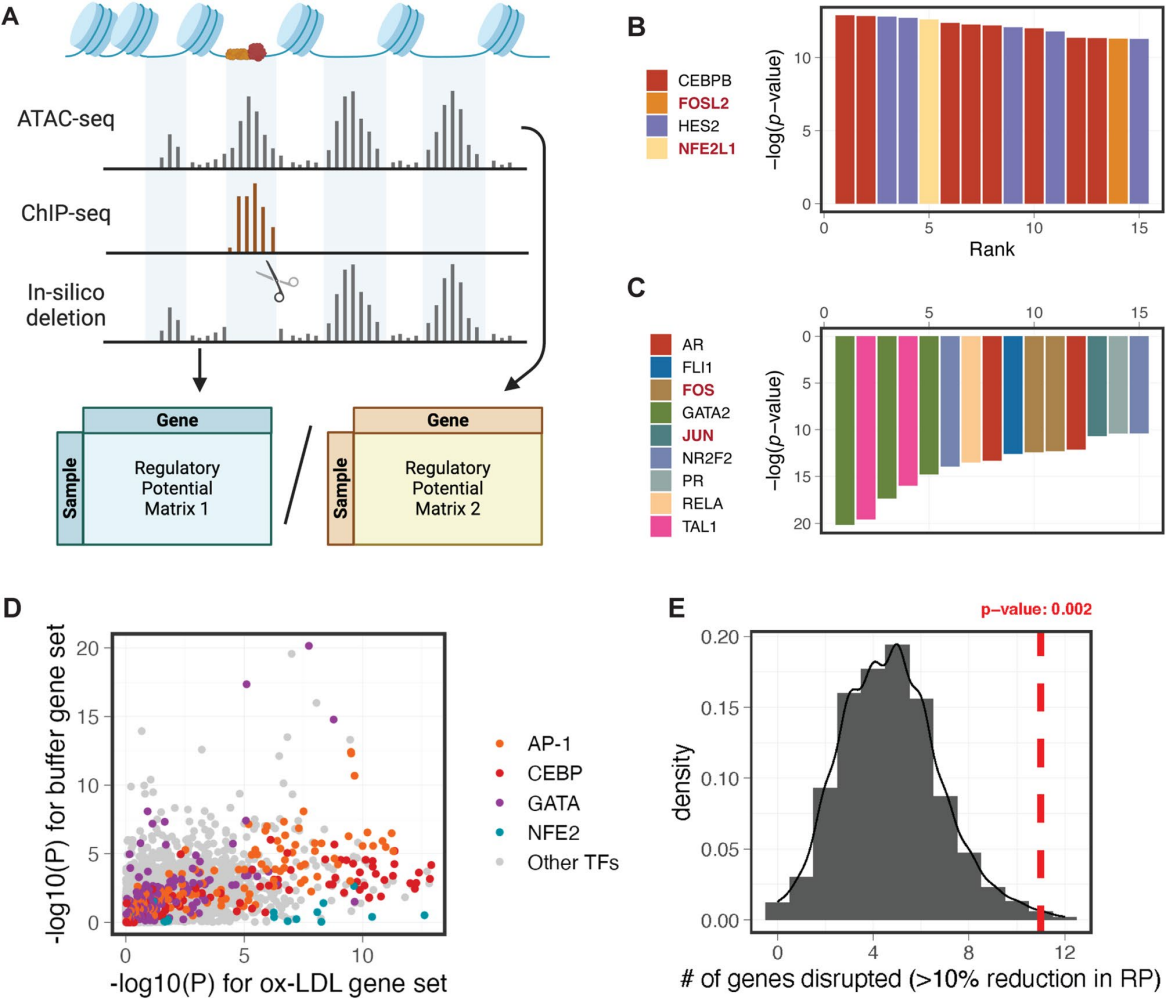
Integrative In-silico deletion (ISD) analysis identifies AP-1 and CEBP family factors as potential regulators underlying the transcriptomic response to ox-LDL. A, Overview of the in-silico deletion analysis: open chromatin signals were obtained from ATAC-seq data; for each transcription factor (TF), potential binding sites were inferred from public ChIP-seq data, and were computationally masked from the endothelial open chromatin landscape. The ATAC-seq signals before and after in-silico deletion were transformed into gene-wise regulatory potentials, and statistical significance was evaluated by comparing the changes in regulatory potential between differentially expressed genes and background genes. B and C, Candidate TFs regulating ox-LDL-induced genes (B) and ox-LDL-repressed genes (C) identified using the ISD analysis in LISA, ranked by significance. Each entry on the x axis represents an independent ChIP-seq experiment. D, ISD p-values of all expressed TFs (*n* = 850 factors from *n* = 5,587 ChIP-seq experiments) for ox-LDL and buffer gene sets. TFs in AP-1, CEBP, GATA and NFE2 families are highlighted. E, ISD of ox-LDL-altered CEBPB-binding sites significantly disrupts the regulatory potential of ox-LDL-induced genes as compared to expression-matched background genes. Genes with > 10% reduction in regulatory potential after the in-silico deletion were considered as being disrupted. The empirical p-value was estimated from the null distribution using *n* = 1000 expression-matched background genes.

We reasoned that gene expression changes in the absence of chromatin accessibility change could result from the re-organisation of transcription factor (TF) binding in pre-existing open chromatin. To investigate the underlying regulators driving the ox-LDL-induced gene expression, we performed in-silico deletion analysis using Lisa^41^integrating both the RNA-seq and ATAC-seq data (Fig. 3A). In brief, Lisa first transforms open chromatin signals (or enhancer signals) into a gene-wise regulatory potentials score, based on the distance to the gene body and promoters. TF binding events, inferred from ChIP-seq datasets, are then deleted in silico from the original open chromatin signals. TFs are identified as potential regulators if their in-silico deletions lead to larger changes in regulatory potentials in the differentially expressed genes, compared to random background genes.

AP-1 factors have been long recognized for their roles in atherogenesis, and have been shown to regulate the oxidative stress response in endothelial cells^42,43^. Consistent with the pathway enrichment analysis (Fig. 2B) and with our previous findings in macrophages^32,44^TFs from the AP-1 and NFE2 family were identified as the top regulators of both ox-LDL-induced genes (Fig. 3B) and ox-LDL-repressed genes in HAECs (Fig. 3C). We also repeated the in-silico deletion analysis with the H3K27ac ChIP-seq data and observed similar results (Figure S5A).

Our previous work has linked the transcription factor CEBPB to the cellular response to ox-LDL in human macrophages^32^. In HAECs, CEBPB displayed the strongest ISD effect on ox-LDL-induced genes (Fig. 3B and D), and was estimated to regulate about 20% of these genes (Figure S5B). The mRNA level of CEBPB, however, did not change after ox-LDL exposure in HAECs (Figure S5C).

To determine whether the regulatory effect of CEBPB is shared across different cell types, we next conducted an ox-LDL-specific in-silico deletion analysis. That is, instead of masking all CEBPB binding signals, we limited the in-silico deletion to regions where CEBPB binding was altered in ox-LDL-exposed macrophages, which represent only 8.7% of the total binding sites (8,718 out of 100,602 peaks, Figure S5D). Compared to randomly selected genes with similar expression levels, ox-LDL-induced genes were significantly more affected by this deletion of altered CEBPB binding sites (Fig. 3E and Figure S5E). Collectively, our results demonstrate a shared ox-LDL-response network between endothelial cells and macrophages that is partly driven by the differential binding of CEBPB.

### Differential transcription factor bindings induced by ox-LDL activates endothelial cell migration

Our in-silico deletion analyses indicate that ox-LDL-induced transcriptomic changes are regulated by differential binding of transcription factors. To further characterise the genomic regions where these TFs act upon ox-LDL exposure, we carried out differential motif footprinting analysis using TOBIAS^45^.

After correcting the Tn5 insertion bias, we identified 2,453 dynamic binding sites (DBSs) where the footprinting score for TF binding changed at least two-fold (*n* = 1,172 and 1,281 sites for ox-LDL-activated and repressed regions, respectively). These DBSs were located across the genome (Figure S6A), and were enriched for H3K27ac signals compared to average open chromatin (Figure S6B).

**Fig. 4:**
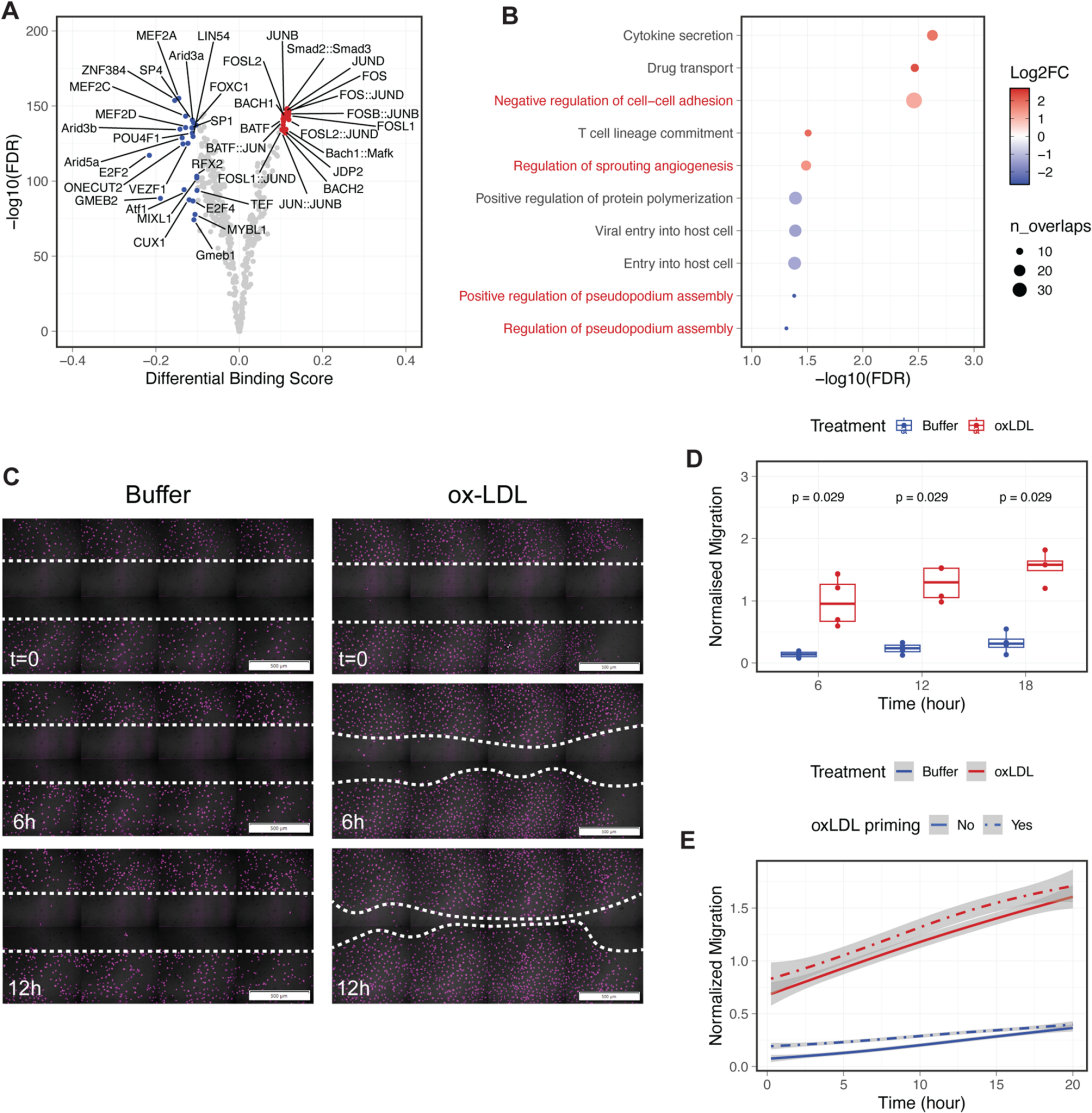
Changes in transcription factor binding activity indicate the activation of endothelial migration pathway in response to ox-LDL. **A**, Volcano plot of transcription factors with genome-wide binding patterns changed after ox-LDL exposure. Significant effect induced or repressed by ox-LDL (FDR < 0.05, $\:\left|{\Delta\:}score\right|>0.1$) are coloured in red and blue, respectively. Only TF expressed in HAECs were included in this analysis. **B**, Predicted functions of the differential binding sites (DBS) using the Genomic Regions Enrichment of Annotations Tool (GREAT). Colour represents log2-fold change and size represents the number of overlapped genes in each annotation. Pathways associated with endothelial migration are highlighted in red. **C**, Representative live-cell fluorescence images of SiR-DNA labelled nuclei at specified time points, with a uniform gap introduced by culture inserts at the start of the assay. Scale bar: 500 μm. **D**, Endothelial migration quantified as the percentage of cells in the gap region normalised to that in buffer-treated cells (*n* = 4 biological replicates). P-values calculated using Wilcoxon rank-sum tests. **E**, Normalised migration of HAECs with or without 24 h of ox-LDL priming. Shaded regions indicate 95% confidence intervals estimated from *n* = 4 biological replicates.

Consistent with previous results, AP-1 and NFE-2 family factors were globally activated in ox-LDL-treated endothelial cells (Fig. 4A), with an increase of 8% for AP-1 factor FOS (Figure S6C). CEBPB was not among the significant hits from footprinting analysis, possibly due to a known discrepancy between its in vivo binding pattern and the reported motif^46^. Overall, these results suggest that ox-LDL treatment triggers a moderate change in TF binding within the already accessible chromatin.

We next performed a pathway enrichment test in DBSs to explore the biological consequence of the observed change in TF binding. Notably, functional annotations related to endothelial migration were highly enriched in genes near both the ox-LDL-activated and ox-LDL-repressed DBSs (Fig. 4B). To experimentally validate this finding, we further performed cell migration assays, and found that ox-LDL exposure in HAECs significantly increased endothelial cell migration (Fig. 4C and D). This experiment was also repeated with cells pre-treated with ox-LDL, and the effect of ox-LDL priming only lasted a few hours after the start of the assay, with no significant difference after 20 h (Fig. 4E). This observation echoes previous results where we detected no global alteration in chromatin accessibility, suggesting a fast and direct endothelial response to ox-LDL exposure.

Together, our computational and experimental results demonstrate that ox-LDL triggers a rapid activation of endothelial migration, potentially mediated by genome-wide epigenomic changes in transcription factor binding.

### Prioritisation of CAD variants using the DBSs in endothelial cells

Endothelial cells are one of the most important arterial cell types that are enriched for the disease heritability of CAD^27,28^. Given that ox-LDL triggers major changes in metabolism, transcriptome and epigenome in HAECs, we hypothesised that the endothelial response to ox-LDL is also genetically linked to human atherosclerosis.

**Fig. 5:**
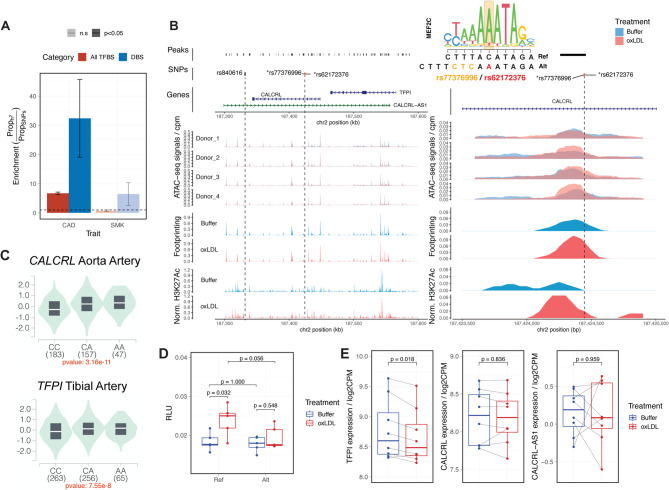
Integrative analysis links CAD risk variants rs62172376 to ***TFPI*** and reveals allelic-specific activation induced by ox-LDL.** A**, Enrichment test for disease heritability in open chromatin regions containing foot prints from TF binding (All TFBS) and dynamic TF binding sites (DBS). Standard errors for enrichment statistics were estimated using s-LDSC. Non-significant enrichment results are plotted in transparent. Enrichment for heritability of smoking behaviour (SMK) is shown to the right as a negative control. GWAS summary statistics were obtained from GWAS Catalog using study ID GCST005194 ^24^, and GCST007327 ^51^ for CAD and SMK, respectively. **B**, The regulatory landscape at the prioritised *CALCRL*/*TFPI* locus. The tagged variant rs840616 is marked in grey and two candidate causal variants rs77376996 and rs62172376 are marked in red. Tracks (from top to bottom) showing: ATAC-seq peaks (peaks), CAD-associated variants (SNPs), genomic annotations (Genes), ATAC-seq signals aggregated by biological donors, Motif footprinting score, and enhancer signals from H3K27ac ChIP-seq aggregated by treatment. Higher resolution coverage plots centred on the risk variant are plotted in the right panel. **C**, Violin plot showing the allele-specific effect of the haplotype on the expression of *CALCRL* and *TFPI* in human artery based on the GTEx data. Samples are grouped by the genotype at the SNP rs62172376. **D**, Dual Luciferase reporter assay of the 150 bp dsDNA construct containing each haplotype. HAECs were transfected with the indicated plasmid, treated with or without ox-LDL for 24 hours and lysed for luciferase activity measurement (*n* = 5 replicates per group). Signals normalised to the Renilla luciferase activity and reported as relative light units (RLU). P-values calculated with Wilcoxon tests. **E**, Normalised gene expression (log2 count-per-million-reads) of *TFPI*, *CALCRL* and *CALCRL-AS1* in unexposed and ox-LDL-exposed HAECs (*n* = 4 biological replicates with 2 technical repeats per donor). P-values were calculated using the quasi-likelihood test in edgeR with a linear mixed model controlling both the donor and batch effect.

To test this hypothesis, we performed an enrichment test for CAD heritability in dynamic TF binding sites. Similar to previous reports, endothelial open chromatin regions that contain all the TF binding sites are enriched for the SNP heritability of CAD (Fig. 5A). This enrichment was not observed for irrelevant neurological traits like smoking behaviour. In addition, we found that the ox-LDL-induced differential binding sites carry 4-times higher per-SNP heritability compared to average binding sites (Fig. 5A), indicating that the genetic risk of CAD is concentrated in the TF binding sites altered by ox-LDL exposure.

Of the 330 independent risk loci that reach the genome wide significance threshold (*p* < 5 × 10^−8^) in genome-wide association studies (GWAS)^25,26^5 contain variants that reside in dynamic binding sites (Table 3). The risk locus at chromosome 9 (9p21.3) has been extensively characterised using knockout mouse models^47^although no eQTL signals were found at the locus in human endothelial cells and vascular smooth muscle cells.

The *PLPP3* gene has been linked to endothelial barrier function in various experimental models^48,49^and a SNP at the *PLPP3* locus was found in an endothelial-specific enhancer reported in a previous variant prioritisation study^22^. Here, we found that another risk variant rs56170783 at this locus overlaps an ox-LDL-induced TF binding site, and displays allelic imbalance in ATAC-seq signals from the heterozygous donor (Figure S7A and B).

**Table 3:** Prioritised CAD-risk variants in ox-LDL-induced DBSs.

Rsid	Chr	Pos	Ref	Alt	DBS	Annotation
rs56170783	chr1	56,550,459	A	C	chr1-56549815–56550830	PLPP3 intron
rs17048367	chr1	218,660,548	A	G, T	chr1-218660506–218661540	Intergenic region
rs7068966	chr10	12,235,993	C	G, T	chr10-12234966–12236001	CDC123 intron
rs77376996	chr2	187,424,445	T	TCTC, TCTCA	chr2-187423908–187424925	CALCRL intron
rs62172376	chr2	187,424,447	C	A	chr2-187423908–187424925	CALCRL intron
rs1537372	chr9	22,103,184	G	A, T	chr9-22103132–22104146	CDKN2B intron
rs1537373	chr9	22,103,342	T	A, G	chr9-22103132–22104146	CDKN2B intron
rs1333042	chr9	22,103,814	A	C, G	chr9-22103132–22104146	CDKN2B intron

### Prioritised variants at the *CALCRL*/*TFPI* locus

At the *CALCRL*/*TFPI* locus, a genetic variant rs840616 has been associated with CAD risks ($\:\beta\:$=0.0402) in European populations^24^. In linkage disequilibrium with this lead variant (r^2^ = 0.81), two closely-linked variants rs77376996 and rs62172376 overlapped with an intronic DBS in the *CALCRL* gene (Fig. 5B). Although the increase in chromatin accessibility at this site was not significant after FDR correction, our motif footprinting analysis indicates a more than two-fold increase in TF binding activity (Fig. 5B). This ox-LDL-induced activation is further supported by the substantial increase in H3K27ac signals in adjacent histones (Fig. 5B).

There are two protein-coding genes *CALCRL* and *TFPI* expressed at this locus, whose transcription start sites are 24 kb and 141 kb downstream of the SNP rs62172376, respectively. This locus also contains a long non-coding RNA *CALCRL-AS1* that is antisense to *CALCRL* and *TFPI*. The protective haplotype (rs62172376-A) contains a MEF2 binding site (Fig. 5B) and in the GTEx database is linked to higher expression of both genes *CALCRL* and *TFPI* in human artery (Fig. 5C).

The allele-specific activation in ox-LDL-exposed endothelial cells was confirmed using dual luciferase reporter assays, where the regulatory element containing the risk reference haplotype displays higher enhancer activity after ox-LDL exposure (Fig. 5D). Interestingly, despite being closer to the DBS, *CALCRL* did not exhibit an ox-LDL-induced change in expression (Fig. 5E). In fact, *TFPI* is the only gene at this locus that was differentially regulated by ox-LDL (log2FC=−0.12, *p* = 0.018; Fig. 5E). These results indicate that *TFPI* but not *CALCRL* is more likely to be the potential causal gene at the CAD risk locus containing rs62172376 which may protect endothelial cells from ox-LDL.

Of note, a closer inspection of molecular QTL signals highlights an alternative splicing event of the anti-sense non-coding RNA *CALCRL-AS1* associated with rs62172376 in human coronary artery (Figure S8A and B). Specifically, we found that the last intron of *CALCRL-AS1* was partially retained in endothelial samples carrying the protective haplotype (Figure S8A). This intron is anti-sense to the promoter sequence of *TFPI*, and its usage positively correlated with the expression of *TFPI* (Figure S8C), but not *CALCRL* (Figure S8D) in HAECs.

Together, our integrative analyses pinpoint a causal variant rs62172376 at the *CALCRL*/*TFPI* locus, prioritise the gene *TFPI* as the target gene, and capture a gene-environment interaction underlying the cellular response to ox-LDL in endothelial cells.

## Discussion

The initial exposure of endothelial cells to ox-LDL marks one of the earliest pathological events in atherosclerosis. In this study, we offer the first comprehensive transcriptomic and epigenomic characterisation of the response to ox-LDL in primary human aortic endothelial cells. Our unbiased genome-wide investigation confirms the involvement of established genes and pathways in the endothelial cell response to ox-LDL. Rather than showing an inflammatory endothelial cell activation in response to oxLDL, we demonstrate an overall non-inflammatory phenotype, with systemic changes in the transcriptome and cellular metabolism. By integrating multi-omics sequencing data, we are able to identify the TFs that drive the ox-LDL response, which features a transcriptional regulatory network that is largely shared with human macrophages. The redistribution of these TFs also activates endothelial-specific pathways, including migration. Crucially, we demonstrate that changes in TF binding induced by ox-LDL are enriched for CAD heritability, and can be utilised as a leverage point to prioritise causal risk variants involved in the endothelial response to atherogenic lipids.

In HAEC, exposure to ox-LDL reduced mitochondrial respiration as previously reported^51^. Assays of glycolytic rate demonstrated that ox-LDL also induces a significant increase in glycolysis as the basis of energy metabolism. These findings differ from those seen with ox-PAPC, a widely studied synthesised component of ox-LDL, which has been reported to increase the respiration and metabolic activity in HAECs^23^. In vivo, we demonstrated a robust correlation between human atherosclerotic lesions and the effect of ox-LDL, but not for ox-PAPC. Clearly, the proatherogenic effect of ox-LDL is a combination of complex, sometimes opposing influences from individual components. Studying one single element within the ox-LDL mixture, despite the increased biochemical specificity and the ease of preparation, may miss the whole picture and so could lead to misleading conclusions.

With a comprehensive genome-wide footprinting analysis, we discovered an activating effect of ox-LDL on endothelial migration. This finding was functionally validated using live-cell microscopy. It is worth noting that contrasting phenotypes have been reported in different endothelial lines at ox-LDL concentrations ranging from 1 to 50 ug/mL^52–54^. In HUVECs, ox-LDL stimulates migration at 5 ug/mL, but inhibits it at 40 ug/mL^52^. On a molecular level, the impaired migration capacity was linked to the dephosphorylation of Akt in HUVECs^53^. However, increased Akt phosphorylation has been reported in HAECs after ox-LDL treatment^55^. Given that HUVECs are venous cells derived from immune-privileged foetal tissue, they may be less tolerant to the oxidative stress compared to adult arterial endothelium. This could also account for the absence of endothelial activation phenotypes in our model using aortic endothelial cells. Nevertheless, we show that the non-inflammatory response to ox-LDL, which drives the migration phenotype, is significantly enriched for disease heritability. In other words, this supports the ox-LDL-induced in vitro migration phenotype being causally involved in the in vivo progression of human atherosclerosis.

Since many non-coding genetic variants influence disease risks by altering the function of regulatory DNA^56^, profiling the colocalization of regulatory regions with genetic risk variants in LD offers a powerful approach to decipher the causal variants and their target genes^57^. The prioritised risk locus around the *CALCRL* and *TFPI* gene is particularly interesting, as only one of the two coding genes is differentially expressed by ox-LDL exposure. The dynamic binding site containing the risk reference haplotype displayed significant activation after ox-LDL treatment, while the protective haplotype remained unchanged. This change in enhancer activity was not found under basal conditions, which may explain why rs62172376 was missed in previous variant prioritisation studies. In fact, our results illustrate a context-specific regulatory effect at this risk locus, underscoring the necessity to adapt experimental models with disease-relevant stimuli in the functional fine-mapping of GWAS results.

There are inevitably limitations with any model, but it is not possible to undertake experiments such as directly exposing cells to ox-LDL in a controlled manner in vivo in humans. Our findings suggest ox-LDL alone does not trigger an inflammatory endothelial phenotype, but it remains possible that in the in vivo disease situation, complex combinations of factors may result in different effects and, for example, ox-LDL has been reported to exacerbate the inflammatory phenotypes in endothelium that has undergone TNF-α- induced damage^14^bacterial infection^58^or overexpression of ox-LDL receptor 1^17^. Indeed, we observed changes in chromatin accessibility at cytokine-related transcription factor binding sites, which could indicate that ox-LDL primes endothelial cells such that they are poised for inflammatory stimulation. Future functional studies could explore the transcriptional regulatory network underlying the ox-LDL response in ECs, especially at genetic risk loci and at the protein expression level.

Overall, this study provides a comprehensive understanding of the transcriptional regulation of ox-LDL response in human endothelial cells. Our multi-omics data lays a solid foundation for future research exploring the interactions between the genetic and environmental component of CAD.

## Materials and methods

### Cell culture

Primary human aortic endothelial cells (HAECs) were obtained from Promocell (Promocell, Heidelberg, Germany) and expanded at passage 2 before usage. HAECs were maintained in Endothelial Cell Growth Medium MV2 (Promocell, Heidelberg, Germany) supplemented with 100 units/mL penicillin and 100 ug/mL streptomycin. Cells were assayed at ~ 80% confluency between passage 4 to passage 7. The catalogue numbers for the four HAEC lines used in this study are: #4082102.16, #422Z037, #431Z002.3, #452Z031.1, and additional demographic information about the biological donors are listed in Supplementary Table S1.

### Cell treatments

LDL was purified from human plasma by isopycnic ultracentrifugation, then oxidized overnight using 25 μm CuCl_2_, as described previously^44^. DPBS solution (Thermo Scientific, Waltham, MA) from the last round of dialysis was used as the control buffer. The concentration of ox-LDL was determined using the BCA protein assay (Thermo Scientific). Unless otherwise specified, cells were treated with a final concentration of 50 ug/mL of ox-LDL or an equal volume of control buffer for 48 h. For immunofluorescence studies, LDL was labelled overnight at 37⁰C with 300 ug DiI per mg LDL, before purification by ultracentrifugation and oxidization using CuCl_2_.

### Flow cytometry

Before the assay, HAECs were treated with 50 ug/mL of DiI-labelled ox-LDL or control buffer, or 10 ug/mL recombinant human TNF alpha protein (ab9642, Abcam) for 24 h. Cells were detached using StemPro Accutase solution (Thermo Scientific) and re-suspended in ice-cold FACS buffer (PBS, 1% BSA and optional 1% azide). Primary antibody staining was carried out in the dark at 4⁰C for 30 min with the following antibody dilutions in FACS buffer: 1:200 ICAM-1 (353102, Biolegend), 1:500 VCAM-1 (14–1069-82, Invitrogen). Second staining was performed with 1:1000 Anti-Mouse-IgG-647 (A-21240, Invitrogen) at 4⁰C for 30 min. Zombie Violet (423113, Biolegend) was included as a viability dye in a final dilution of 1:200. Data was exported to FlowJo 10 (FlowJo LLC, Ashland, USA) for quantitative analysis.

### Immunofluorescence imaging

For fixed cell imaging, HAECs were seeded at 15,000 cells/well in 8-well ibiTreat µ-slides (Ibidi) and assayed at ~ 80% confluency before treatment with 50 ug/mL DiI-labelled ox-LDL or control buffer. Cells were fixed at room temperature for 15 min with 4% PFA in PBS, followed by permeabilization with 0.1% Triton X-100 (Thermo Scientific) and 0.2% Tween-20 (Roche) in PBS. Fixed cells were incubated in blocking buffer (1% BSA, 2% FCS, 0.3 M glycine and 0.1% Tween-20 in PBS) at room temperature for 1 h. Cytoskeleton was staining with 1:500 tubulin-AF488 (clone DM1A, eBioscience) and nuclei were counterstained with Hoechst 33,342 (Santa Cruz Biotechnology) diluted 1:10,000 in PBS. Stained cells were imaged on a LSM 900 with Airyscan2 in confocal mode (Zeiss).

For the migration assay, cell culture plates with 2-well silicone inserts (80241, Ibidi) were used to create a 500 μm cell-free gap for uniform imaging of endothelial migration. Prior to the assay, HAECs were seeded at the density of 10,000 cells per culture insert well and were starved in supplement-depleted EC culture medium containing 50ug/mL ox-LDL or control buffer overnight. Before the assay, cells were pre-stained with 300 nM SiR-DNA (CY-SC007, Universal Biologicals) for at least 2 h. Culture inserts were carefully lifted using a sterile tweezer, and cells were gently washed with sterile PBS twice. Live cells were then imaged every 15 min for 24 h in assay media containing 300 nM SiR-DNA and 50 ug/mL ox-LDL or control buffer on the Olympus SpinSR SoRa and ScanR High Content system (Olympus). The location of nuclei was recovered using arivis Vision4D version 4.1 (Zeiss), and the migration rate was defined as the percentage of nuclei overlapping the initial gap region. Migration rates were normalised to the buffer group within paired samples to mitigate any donor effect.

### Seahorse

Metabolic measurements of HAEC were performed with Seahorse XF Mito Stress Test and XF Glycolytic Rate Assay kits using a Seahorse XFe96 Analyzer (Agilent Technologies, Santa Clara, US). 50,000 endothelial cells were seeded into the Seahorse 96-well plates and were treated with 50ug/mL oxLDL or control buffer 24 h prior to the assay. For mito stress test, 2 μm of Oligomycin, 1 μm of FCCP and 0.5 μm of Rotenone/Antimycin A were used based on a pilot titration experiment; For glycolytic rate, 0.5 μm of Rotenone/Antimycin A and 50mM of 2-DG were used. Results were analysed using Seahorse Wave Desktop (Agilent).

### Library preparation for high-throughput sequencing

Total RNA from HAECs was purified using RNeasy Micro Kit (Qiagen) after TRIzol and chloroform extraction according to the manufacturer’s protocol. The quality of RNA samples was analysed using a TapeStation 2200 with High Sensitivity RNA ScreenTape (Agilent Technologies, Santa Clara, US). Samples with high integrity (RIN score > 9) were selected for PolyA-enrichment and TruSeq library preparation (Illumina). Each sample was sequenced to a target depth of 37.5 million read pairs using a NovaSeq 6000 device (Illumina).

ATAC-seq libraries were prepared according to the Omni-ATAC protocol^59^. In brief, nuclei were isolated by incubation with ATAC-Resuspension Buffer (ATAC-RSB) containing 0.1% NP40 (Roche, Basel, Switzerland), 0.1% Tween-20 (Roche, Basel, Switzerland) and 0.01% Digitonin (Promega, Madison, WI) on ice for 3 min, and were washed with 1 mL of ATAC-RSB containing 0.1% Tween-20. Tn5 tagmentation reaction was performed at 37 °C for 30 min using the Nextera kit (Illumina). ATAC-seq libraries were then PCR amplified using NEBNext High-Fidelity PCR master mix (New England Biolabs, Ipswich, US) and index primers from Nextera Kit (Illumina), and analysed using a TapeStation 2200 with High Sensitivity D1000 ScreenTape (Agilent). Libraries showing successful transposition were sequenced to a target depth of 112.5 million read pairs using a NovaSeq 6000 device (Illumina).

ChIP-seq was performed using the MAGnify Chromatin Immunoprecipitation System (ThermoFisher Scientific) following the manufacturer’s instructions. HAECs were resuspended in 1% formaldehyde at room temperature for 10 min, and the cross-linked chromatins were fragmented using a Bioruptor (Diagenode) with 16 cycles of 30 s on and off. 500,000 cells per IP were incubated with 100uL Dynabeads conjugated with 2 ul of Anti-H3K27ac (ab472, Abcam) or Anti-Rabbit-IgG control (Thermo Scientific) at 4 °C overnight. After reverse crosslinking, ChIP-ed DNA was purified using MinElute Kit (Qiagen) followed by TruSeq library preparation (Illumina). were sequenced to a target depth of 40 million read pairs using a Hi-Seq 2500 device (Illumina).

For all bulk sequencing libraries, read qualities were checked using FastQC^60^. When adapter contamination was detected, the 3’ end of affected reads was trimmed using NGMerge^61^.

### RNA-seq data analysis

Adaptor-trimmed reads were first aligned to the reference human genome using the splice-aware STAR aligner^62^. Human genome assembly GRCh38.p13 and gene annotation Release 43 were downloaded from the GENCODE site and were indexed using STAR. SAMtools^63^ was used to filter out unmapped or multi-mapping reads, and gene-wise count matrices were generated using featureCounts of the Subread package^64^. Differential expression analysis was performed in R using edgeR^65^. Genes with low expression (min.count < 50 in more than half of the samples) were filtered out, and the remaining count matrices were normalised by sequencing depth and by the Trimmed Mean of M-values (TMM) method. Differential gene expression between treatment groups was tested using the quasi-likelihood test, with a generalized linear model controlling for experimental batches and biological donors. The test statistics were adjusted using the Benjamini-Hochberg (B-H) method and genes with adjusted p-values < 0.05 were considered as differentially expressed. Pathway enrichment analyses were performed using the XGR package^66^with functional annotations collected from MsigDB and KEGG. The differential expression statistics of all tested genes (*n* = 13,424) are provided in Supplementary Table S2.

Single-cell RNA-seq profiles of human carotid atherosclerotic plaque and adjacent control tissue were retrieved from Gene Expression Omnibus using the accession number GSE159677 ^67^, as part of our recent meta-analysis in^32^. Endothelial cells were selected based on gene expression of *PECAM1*, *VWF* and *PLVAP*, and were aggregated into pseudo-bulk population per sample. Differential expression tests were performed using edgeR with the quasi-likelihood test, with donor information included as a covariate. The Rank-Rank Hypergeometric Overlap tested were conducted in R using the package RRHO^40^and genes were ranked by sign(log-fold-change)$\:\times\:$log_10_(P).

### ATAC-seq data analysis

Adaptor-trimmed reads were aligned to the hg38 reference genome using Bowtie2 with --very-sensitive settings^68^. PCR duplicates were marked using Picard, and were further removed from downstream analysis together with mitochondrial reads using SAMtools. Post-alignment QC was performed as instructed by the ENCODE ATAC-seq processing pipeline, and all libraries passed the threshold of Non-Redundant Fraction > 0.9, PCR Bottleneck Coefficients-1 > 0.9, PCR Bottleneck Coefficients-2 > 3, and Transcription Start Site Enrichment score > 7.

For downstream analysis, reads were trimmed using BEDTools^69^ to retain only the 9-bp Tn5 cutting sites at both ends of the DNA fragments. A consensus peak set was called using MACS3^70^ combining all sequenced samples, with peaks overlapping the ENCODE blacklisted region being removed before read counting in Subread. Differential accessibility analysis was performed using edgeR, as described above. A window-based differential accessibility test was also performed by counting reads within 100 bp sliding windows across the genome using csaw^71^.

To determine the donor genotype, aligned ATAC-seq reads were aggregated by sample and genotyped using the GATK (v4.1.7.0) germline short variant discovery pipeline^72^. PCR duplicates were marked with MarkDuplicates. Base quality scores were recalibrated against dbSNP155 known sites, and variants were called with HaplotypeCaller, combined using GenomicsDBImport, and genotyped with GenotypeGVCFs. Genotyping results from SNPs in perfect LD (r^2^ = 1 in 1000G EUR samples) were merged.

### ChIP-seq data analysis

As described above, adaptor-trimmed reads were aligned to the hg38 reference genome using Bowtie2 followed by PCR duplicates removal using Picard. For H3K27ac ChIP-seq, aligned bam files were merged with subsampling per treatment group. Aggregated ChIP-seq signals were computed over 100 bp bins and normalized by sequencing depth using the bamCoverage from DeepTools, with 150 bp extension of each fragment. For function annotation of dynamic binding sites (DBSs), ChIP-seq reads were counted per 100 bp bin for each 4 kb window centred at each DBS, normalized by sequencing depth, and smoothed using a generalized additive model implemented through ggplot2. The macrophage CEBPB ChIP-seq dataset was generated previously and retrieved from GEO (GSE54975)^44^. A consensus peak set was called using MACS3 with all samples, and peaks overlapping the ENCODE blacklisted region were removed. Differential binding tests were performed in edgeR, with both donor and treatment included as covariates in the quasi-likelihood test. Peaks with p-values lower than 0.05 were retained (*n* = 8,718) for ox-LDL specific in-silico deletion analysis.

### In-silico deletion analysis

In-silico deletion (ISD) tests were performed using the “FromCoverage” function of the Lisa package^41^ with 500 background genes and the default ‘enhanced_10k’ regulatory potential model. The consensus open chromatin profile from ATAC-seq or the consensus enhancer profile from H3K27ac ChIP-seq was used to compute the background regulatory potential. For the ISD of ox-LDL-specific CEBPB bindings, fragments overlapping altered CEBPB binding sites were removed using BEDTools, and the gene-wise regulatory potentials were calculated using the “genescore” function implemented through the MAESTRO package^73^; empirical null distribution was estimated from *n* = 1000 sets of expression-matched background genes.

### Differential motif footprinting analysis

TOBIAS^45^ was used to analyse the foot print signatures in ATAC-seq data. In brief, the merged 9-bp consensus bam file was processed using ATACorrect function in TOBIAS to correct for the intrinsic bias in Tn5-cut sites; Footprinting scores were calculated at base-pair resolution within open chromatin peaks using ScoreBigwig; differential foot printing was then conducted using BINDetect, with transcription factor (TF) motifs downloaded from JASPAR core 2018^74^. For ontology enrichment and heritability analysis, differential TF binding sites was extended 500 bp at each end; the enrichment for functional annotations was tested using GREAT v4^75^.

### GWAS and QTL SNPs

GWAS statistics were curated by NHGRI-EBI Catalog and downloaded from the UCSC hg38 database (April 2022). We identified SNPs with significant genome-wide association (*p* < 5 × 10^−^), pruned the list (r^2^ > 0.1 identified by 1000 Genome Project phase 3 in EUR samples, hg38 build) to remove redundant loci, and then pooled all SNPs in high LD within each locus (r^2^ > 0.8 in EUR samples) to establish a set of trait-associated variants. Only dbSNP common variants (MAF > 1%, dbSNP155) were considered in this study. LD information was extracted using PLINK v1.90b6.26^76^. eQTL SNPs were obtained from GTEx v8 (Aorta Artery, Coronary Artery and Tibial Artery)^77^ at hg19 coordinates were converted to hg38 using annotations from NCBI dbSNP Build 155.

### Heritability analysis

LDSC v1.01 was used to partition CAD heritability and test the enrichment for DBSs as described^78,79^. The standard errors of enrichment statistics and regression coefficients are computed using a block jack-knife (*n* = 1,000)^78^. The full summary statistics for CAD used in this study were downloaded from the GWAS Catalog, study ID GCST005194 ^24^. Smoking behaviour was included as a negative control and its summary statistics were retrieved using with the ID GCST007327^50^.

### Dual Luciferase Assay

The 150-bp enhancer element containing either the reference or the alternative haplotype centred at rs62172376 was synthesized (GenScript Biotech, Piscataway, US) and cloned into empty pGL4.27 Firefly luciferase reporter (Promega, Fitchburg, WI). Successful cloning was verified by sequencing prior to transfection. For dual luciferase assay, 1 ug pGL4.27 Firefly luciferase reporter plasmid and 0.02 ug pRL-SV40 Renilla luciferase plasmid were co-transfected using ViaFect Transfection Reagent (Promega) in a 1:6 ratio per well in 12-well plates. HAECs were treated with 50 ug/mL ox-LDL or buffer 24 h post transfection for another 24 h, and were lysed for luciferase assay using the Dual Luciferase Reporter Assay System (Promega). Luciferase activities were measured using a CLARIOstar Microplate Reader (BMG Labtech, Ortenberg, Germany), and Firefly luciferase activity was normalized to Renilla luciferase activity as relative light units.

### Statistical analysis

Data was reported using mean ± standard error of the mean (SEM), or using boxplots displaying the median and quartiles. When normality could not be assumed, Wilcoxon rank-sum tests were used to evaluate the significance of differences between two experimental groups. In experiments that had ≥ 3 experimental groups, one-way analysis of variance (ANOVA) was employed with Dunnett’s post-hoc tests. Unless otherwise specified, all statistical analyses were performed in R (version 4.2.0).

## Electronic supplementary material

Below is the link to the electronic supplementary material.

Supplementary Material 1

Supplementary Material 2

## Data Availability

Sequencing data that support the findings of this study have been deposited in the National Center for Biotechnology Information Sequence Read Archive (NCBI-SRA) under accession number PRJNA1145858, and are available at the following URL: https://www.ncbi.nlm.nih.gov/bioproject/PRJNA1145858.

## Declarations

Sequencing data that support the findings of this study have been deposited in the National Center for Biotechnology Information Sequence Read Archive (NCBI-SRA) under accession number PRJNA1145858, and are available at the following URL: https://www.ncbi.nlm.nih.gov/bioproject/PRJNA1145858.

## References

[1] Song, P. & Zou, M. H. Redox regulation of endothelial cell fate. *Cell. Mol. Life Sci.* **71**, 3219–3239. 10.1007/s00018-014-1598-z (2014). 24633153 10.1007/s00018-014-1598-z PMC4134393

[2] Davignon, J. & Ganz, P. Role of endothelial dysfunction in atherosclerosis. *Circulation* **109**, III27–32. 10.1161/01.CIR.0000131515.03336.f8 (2004). 15198963 10.1161/01.CIR.0000131515.03336.f8

[3] Chevre, R. et al. High-resolution imaging of intravascular atherogenic inflammation in live mice. *Circ. Res.* **114**, 770–779. 10.1161/CIRCRESAHA.114.302590 (2014). 24366169 10.1161/CIRCRESAHA.114.302590

[4] Gimbrone, M. A. Jr. & Garcia-Cardena, G. Endothelial cell dysfunction and the pathobiology of atherosclerosis. *Circ. Res.* **118**, 620–636. 10.1161/CIRCRESAHA.115.306301 (2016). 26892962 10.1161/CIRCRESAHA.115.306301 PMC4762052

[5] Pober, J. S. & Sessa, W. C. Evolving functions of endothelial cells in inflammation. *Nat. Rev. Immunol.* **7**, 803–815. 10.1038/nri2171 (2007). 17893694 10.1038/nri2171

[6] Hansson, G. K. Inflammation, atherosclerosis, and coronary artery disease. *N Engl. J. Med.* **352**, 1685–1695. 10.1056/NEJMra043430 (2005). 15843671 10.1056/NEJMra043430

[7] Virmani, R., Kolodgie, F. D., Burke, A. P., Farb, A. & Schwartz, S. M. Lessons from sudden coronary death: a comprehensive morphological classification scheme for atherosclerotic lesions. *Arterioscler. Thromb. Vasc Biol.* **20**, 1262–1275. 10.1161/01.atv.20.5.1262 (2000). 10807742 10.1161/01.atv.20.5.1262

[8] Jiang, H. et al. Mechanisms of oxidized LDL-Mediated endothelial dysfunction and its consequences for the development of atherosclerosis. *Front. Cardiovasc. Med.* **9**, 925923. 10.3389/fcvm.2022.925923 (2022). 35722128 10.3389/fcvm.2022.925923 PMC9199460

[9] Li, D. & Mehta, J. L. Antisense to LOX-1 inhibits oxidized LDL-mediated upregulation of monocyte chemoattractant protein-1 and monocyte adhesion to human coronary artery endothelial cells. *Circulation* **101**, 2889–2895. 10.1161/01.cir.101.25.2889 (2000). 10869259 10.1161/01.cir.101.25.2889

[10] Sohrabi, Y. et al. OxLDL-mediated Immunologic memory in endothelial cells. *J. Mol. Cell. Cardiol.* **146**, 121–132. 10.1016/j.yjmcc.2020.07.006 (2020). 32726647 10.1016/j.yjmcc.2020.07.006

[11] Zhu, H. et al. Ox-LDL plays dual effect in modulating expression of inflammatory molecules through LOX-1 pathway in human umbilical vein endothelial cells. *Front. Biosci.* **10**, 2585–2594. 10.2741/1722 (2005). 15970520 10.2741/1722

[12] Takei, A., Huang, Y. & Lopes-Virella, M. F. Expression of adhesion molecules by human endothelial cells exposed to oxidized low density lipoprotein. Influences of degree of oxidation and location of oxidized LDL. *Atherosclerosis* **154**, 79–86. 10.1016/s0021-9150(00)00465-2 (2001). 11137085 10.1016/s0021-9150(00)00465-2

[13] Dwivedi, A., Anggard, E. E. & Carrier, M. J. Oxidized LDL-mediated monocyte adhesion to endothelial cells does not involve NFkappaB. *Biochem. Biophys. Res. Commun.* **284**, 239–244. 10.1006/bbrc.2001.4955 (2001). 11374896 10.1006/bbrc.2001.4955

[14] Khan, B. V., Parthasarathy, S. S., Alexander, R. W. & Medford, R. M. Modified low density lipoprotein and its constituents augment cytokine-activated vascular cell adhesion molecule-1 gene expression in human vascular endothelial cells. *J. Clin. Invest.* **95**, 1262–1270. 10.1172/JCI117776 (1995). 7533787 10.1172/JCI117776 PMC441465

[15] Chen, C. & Khismatullin, D. B. Oxidized low-density lipoprotein contributes to atherogenesis via co-activation of macrophages and mast cells. *PLoS One*. **10**, e0123088. 10.1371/journal.pone.0123088 (2015). 25811595 10.1371/journal.pone.0123088 PMC4374860

[16] Amberger, A. et al. Co-expression of ICAM-1, VCAM-1, ELAM-1 and Hsp60 in human arterial and venous endothelial cells in response to cytokines and oxidized low-density lipoproteins. *Cell. Stress Chaperones*. **2**, 94–103. 10.1379/1466-1268(1997)002%3C0094:ceoive%3E2.3.co;2 (1997). 9250400 10.1379/1466-1268(1997)002<0094:ceoive>2.3.co;2 PMC312986

[17] Mattaliano, M. D. et al. LOX-1-dependent transcriptional regulation in response to oxidized LDL treatment of human aortic endothelial cells. *Am. J. Physiol. Cell. Physiol.* **296**, C1329–1337. 10.1152/ajpcell.00513.2008 (2009). 19279231 10.1152/ajpcell.00513.2008

[18] Watson, A. D. et al. Structural identification by mass spectrometry of oxidized phospholipids in minimally oxidized low density lipoprotein that induce monocyte/endothelial interactions and evidence for their presence in vivo. *J. Biol. Chem.* **272**, 13597–13607. 10.1074/jbc.272.21.13597 (1997). 9153208 10.1074/jbc.272.21.13597

[19] Chu, L. H. et al. The oxidized phospholipid OxPAPC protects from septic shock by targeting the non-canonical inflammasome in macrophages. *Nat. Commun.* **9**, 996. 10.1038/s41467-018-03409-3 (2018). 29520027 10.1038/s41467-018-03409-3 PMC5843631

[20] Appleton, B. D., Palmer, S. A., Smith, H. P., Stephens, L. E. & Major, A. S. Oxidized phospholipid OxPAPC alters regulatory T-Cell differentiation and decreases their protective function in atherosclerosis in mice. *Arterioscler. Thromb. Vasc Biol.* **43**, 2119–2132. 10.1161/ATVBAHA.123.319674 (2023). 37675632 10.1161/ATVBAHA.123.319674 PMC10720352

[21] Romanoski, C. E. et al. Network for activation of human endothelial cells by oxidized phospholipids: a critical role of Heme Oxygenase 1. *Circ. Res.* **109**, e27–41. 10.1161/CIRCRESAHA.111.241869 (2011). 21737788 10.1161/CIRCRESAHA.111.241869 PMC3163234

[22] Hogan, N. T. et al. Transcriptional networks specifying homeostatic and inflammatory programs of gene expression in human aortic endothelial cells. *Elife* **6**10.7554/eLife.22536 (2017). 10.7554/eLife.22536 PMC5461113 28585919

[23] Hitzel, J. et al. Oxidized phospholipids regulate amino acid metabolism through MTHFD2 to facilitate nucleotide release in endothelial cells. *Nat. Commun.* **9**, 2292. 10.1038/s41467-018-04602-0 (2018). 29895827 10.1038/s41467-018-04602-0 PMC5997752

[24] van der Harst, P. & Verweij, N. Identification of 64 novel genetic loci provides an expanded view on the genetic architecture of coronary artery disease. *Circ. Res.* **122**, 433–443. 10.1161/CIRCRESAHA.117.312086 (2018). 29212778 10.1161/CIRCRESAHA.117.312086 PMC5805277

[25] Aragam, K. G. et al. Discovery and systematic characterization of risk variants and genes for coronary artery disease in over a million participants. *Nat. Genet.* **54**, 1803–1815. 10.1038/s41588-022-01233-6 (2022). 36474045 10.1038/s41588-022-01233-6 PMC9729111

[26] Tcheandjieu, C. et al. Large-scale genome-wide association study of coronary artery disease in genetically diverse populations. *Nat. Med.* **28**, 1679–1692. 10.1038/s41591-022-01891-3 (2022). 35915156 10.1038/s41591-022-01891-3 PMC9419655

[27] Turner, A. W. et al. Single-nucleus chromatin accessibility profiling highlights regulatory mechanisms of coronary artery disease risk. *Nat. Genet.* **54**, 804–816. 10.1038/s41588-022-01069-0 (2022). 35590109 10.1038/s41588-022-01069-0 PMC9203933

[28] Slenders, L. et al. Intersecting single-cell transcriptomics and genome-wide association studies identifies crucial cell populations and candidate genes for atherosclerosis. *Eur. Heart J. Open.* **2**, oeab043. 10.1093/ehjopen/oeab043 (2022). 35174364 10.1093/ehjopen/oeab043 PMC8841481

[29] Gupta, R. M. et al. A Genetic Variant Associated with Five Vascular Diseases Is a Distal Regulator of Endothelin-1 Gene Expression. *Cell* 170, 522–533 e515 (2017). 10.1016/j.cell.2017.06.049 10.1016/j.cell.2017.06.049 PMC5785707 28753427

[30] Konta, A. et al. A functional SNP in FLT1 increases risk of coronary artery disease in a Japanese population. *J. Hum. Genet.* **61**, 435–441. 10.1038/jhg.2015.171 (2016). 26791355 10.1038/jhg.2015.171

[31] Abumrad, N. A. et al. Endothelial cell receptors in tissue lipid uptake and metabolism. *Circ. Res.* **128**, 433–450. 10.1161/CIRCRESAHA.120.318003 (2021). 33539224 10.1161/CIRCRESAHA.120.318003 PMC7959116

[32] Jiang, J. et al. A novel macrophage subpopulation conveys increased genetic risk of coronary artery disease. *Circ. Res.*10.1161/CIRCRESAHA.123.324172 (2024). 38747151 10.1161/CIRCRESAHA.123.324172 PMC11191562

[33] Sawamura, T. et al. An endothelial receptor for oxidized low-density lipoprotein. *Nature* **386**, 73–77. 10.1038/386073a0 (1997). 9052782 10.1038/386073a0

[34] Moore, J. M., Bell, E. L., Hughes, R. O. & Garfield, A. S. ABC transporters: human disease and pharmacotherapeutic potential. *Trends Mol. Med.* **29**, 152–172. 10.1016/j.molmed.2022.11.001 (2023). 36503994 10.1016/j.molmed.2022.11.001

[35] Ade, N. et al. HMOX1 and NQO1 genes are upregulated in response to contact sensitizers in dendritic cells and THP-1 cell line: role of the Keap1/Nrf2 pathway. *Toxicol. Sci.* **107**, 451–460. 10.1093/toxsci/kfn243 (2009). 19033392 10.1093/toxsci/kfn243

[36] Li, Z., Lange, M., Dixon, S. J. & Olzmann, J. A. Lipid quality control and ferroptosis: from concept to mechanism. *Annu. Rev. Biochem.* **93**, 499–528. 10.1146/annurev-biochem-052521-033527 (2024). 37963395 10.1146/annurev-biochem-052521-033527 PMC11091000

[37] Yuan, W. et al. The role of ferroptosis in endothelial cell dysfunction. *Cell. Cycle*. **21**, 1897–1914. 10.1080/15384101.2022.2079054 (2022). 35579940 10.1080/15384101.2022.2079054 PMC9415627

[38] Bai, T., Li, M., Liu, Y., Qiao, Z. & Wang, Z. Inhibition of ferroptosis alleviates atherosclerosis through attenuating lipid peroxidation and endothelial dysfunction in mouse aortic endothelial cell. *Free Radic Biol. Med.* **160**, 92–102. 10.1016/j.freeradbiomed.2020.07.026 (2020). 32768568 10.1016/j.freeradbiomed.2020.07.026

[39] Perera, R. M., Di Malta, C. & Ballabio, A. MiT/TFE family of transcription factors, lysosomes, and Cancer. *Annu. Rev. Cancer Biol.* **3**, 203–222. 10.1146/annurev-cancerbio-030518-055835 (2019). 31650096 10.1146/annurev-cancerbio-030518-055835 PMC6812561

[40] Plaisier, S. B., Taschereau, R., Wong, J. A. & Graeber, T. G. Rank-rank hypergeometric overlap: identification of statistically significant overlap between gene-expression signatures. *Nucleic Acids Res.* **38**, e169. 10.1093/nar/gkq636 (2010). 20660011 10.1093/nar/gkq636 PMC2943622

[41] Qin, Q. et al. Lisa: inferring transcriptional regulators through integrative modeling of public chromatin accessibility and ChIP-seq data. *Genome Biol.* **21**, 32. 10.1186/s13059-020-1934-6 (2020). 32033573 10.1186/s13059-020-1934-6 PMC7007693

[42] Valente, A. J., Irimpen, A. M., Siebenlist, U. & Chandrasekar, B. OxLDL induces endothelial dysfunction and death via TRAF3IP2: Inhibition by HDL3 and AMPK activators. *Free Radic Biol. Med.* **70**, 117–128. 10.1016/j.freeradbiomed.2014.02.014 (2014). 24561578 10.1016/j.freeradbiomed.2014.02.014 PMC4006317

[43] Osto, E. et al. c-Jun N-terminal kinase 2 deficiency protects against hypercholesterolemia-induced endothelial dysfunction and oxidative stress. *Circulation* **118**, 2073–2080. 10.1161/CIRCULATIONAHA.108.765032 (2008). 18955669 10.1161/CIRCULATIONAHA.108.765032

[44] Reschen, M. E. et al. Lipid-induced epigenomic changes in human macrophages identify a coronary artery disease-associated variant that regulates PPAP2B expression through altered C/EBP-beta binding. *PLoS Genet.* **11**, e1005061. 10.1371/journal.pgen.1005061 (2015). 25835000 10.1371/journal.pgen.1005061 PMC4383549

[45] Bentsen, M. et al. ATAC-seq footprinting unravels kinetics of transcription factor binding during zygotic genome activation. *Nat. Commun.* **11**, 4267. 10.1038/s41467-020-18035-1 (2020). 32848148 10.1038/s41467-020-18035-1 PMC7449963

[46] Cohen, D. M., Lim, H. W., Won, K. J. & Steger, D. J. Shared nucleotide flanks confer transcriptional competency to bZip core motifs. *Nucleic Acids Res.* **46**, 8371–8384. 10.1093/nar/gky681 (2018). 30085281 10.1093/nar/gky681 PMC6144830

[47] Kim, J. B. et al. Effect of 9p21.3 coronary artery disease locus neighboring genes on atherosclerosis in mice. *Circulation* **126**, 1896–1906. 10.1161/CIRCULATIONAHA.111.064881 (2012). 22952318 10.1161/CIRCULATIONAHA.111.064881 PMC3608429

[48] Mueller, P. A. et al. Coronary artery disease Risk-Associated Plpp3 gene and its product lipid phosphate phosphatase 3 regulate experimental atherosclerosis. *Arterioscler. Thromb. Vasc Biol.* **39**, 2261–2272. 10.1161/ATVBAHA.119.313056 (2019). 31533471 10.1161/ATVBAHA.119.313056 PMC6812632

[49] Chatterjee, I., Baruah, J., Lurie, E. E. & Wary, K. K. Endothelial lipid phosphate phosphatase-3 deficiency that disrupts the endothelial barrier function is a modifier of cardiovascular development. *Cardiovasc. Res.* **111**, 105–118. 10.1093/cvr/cvw090 (2016). 27125875 10.1093/cvr/cvw090 PMC4909162

[50] Backman, J. D. et al. Exome sequencing and analysis of 454,787 UK biobank participants. *Nature* **599**, 628–634. 10.1038/s41586-021-04103-z (2021). 34662886 10.1038/s41586-021-04103-z PMC8596853

[51] Wang, G. et al. LncRNA FENDRR inhibits ox-LDL induced mitochondrial energy metabolism disorder in aortic endothelial cells via miR-18a-5p/PGC-1alpha signaling pathway. *Front. Endocrinol. (Lausanne)*. **12**, 622665. 10.3389/fendo.2021.622665 (2021). 33912133 10.3389/fendo.2021.622665 PMC8072360

[52] Qiu, J. et al. Coordination of Id1 and p53 activation by oxidized LDL regulates endothelial cell proliferation and migration. *Ann. Biomed. Eng.* **39**, 2869–2878. 10.1007/s10439-011-0382-6 (2011). 21870248 10.1007/s10439-011-0382-6

[53] Chavakis, E. et al. Oxidized LDL inhibits vascular endothelial growth factor-induced endothelial cell migration by an inhibitory effect on the akt/endothelial nitric oxide synthase pathway. *Circulation* **103**, 2102–2107. 10.1161/01.cir.103.16.2102 (2001). 11319202 10.1161/01.cir.103.16.2102

[54] Murugesan, G., Chisolm, G. M. & Fox, P. L. Oxidized low density lipoprotein inhibits the migration of aortic endothelial cells in vitro. *J. Cell. Biol.* **120**, 1011–1019. 10.1083/jcb.120.4.1011 (1993). 8432723 10.1083/jcb.120.4.1011 PMC2200083

[55] Zhang, C. et al. OxLDL induces endothelial cell proliferation via Rho/ROCK/Akt/p27(kip1) signaling: opposite effects of OxLDL and cholesterol loading. *Am. J. Physiol. Cell. Physiol.* **313**, C340–C351. 10.1152/ajpcell.00249.2016 (2017). 28701359 10.1152/ajpcell.00249.2016 PMC5625097

[56] Zhang, F. & Lupski, J. R. Non-coding genetic variants in human disease. *Hum. Mol. Genet.* **24**, R102–110. 10.1093/hmg/ddv259 (2015). 26152199 10.1093/hmg/ddv259 PMC4572001

[57] Cano-Gamez, E. & Trynka, G. From GWAS to function: using functional genomics to identify the mechanisms underlying complex diseases. *Front. Genet.* **11**, 424. 10.3389/fgene.2020.00424 (2020). 32477401 10.3389/fgene.2020.00424 PMC7237642

[58] Vielma, S. A., Mironova, M., Ku, J. R. & Lopes-Virella, M. F. Oxidized LDL further enhances expression of adhesion molecules in Chlamydophila pneumoniae-infected endothelial cells. *J. Lipid Res.* **45**, 873–880. 10.1194/jlr.M300456-JLR200 (2004). 14967815 10.1194/jlr.M300456-JLR200

[59] Corces, M. R. et al. An improved ATAC-seq protocol reduces background and enables interrogation of frozen tissues. *Nat. Methods*. **14**, 959–962. 10.1038/nmeth.4396 (2017). 28846090 10.1038/nmeth.4396 PMC5623106

[60] Andrews, S. FastQC: a quality control tool for high throughput sequence data. Available online at: http://www.bioinformatics.babraham.ac.uk/projects/fastqc (2010)

[61] Gaspar, J. M. NGmerge: merging paired-end reads via novel empirically-derived models of sequencing errors. *BMC Bioinform.* **19**, 536. 10.1186/s12859-018-2579-2 (2018). 10.1186/s12859-018-2579-2 PMC6302405 30572828

[62] Dobin, A. et al. STAR: ultrafast universal RNA-seq aligner. *Bioinformatics* **29**, 15–21. 10.1093/bioinformatics/bts635 (2013). 23104886 10.1093/bioinformatics/bts635 PMC3530905

[63] Li, H. et al. The sequence alignment/map format and samtools. *Bioinformatics* **25**, 2078–2079. 10.1093/bioinformatics/btp352 (2009). 19505943 10.1093/bioinformatics/btp352 PMC2723002

[64] Liao, Y., Smyth, G. K. & Shi, W. The Subread aligner: fast, accurate and scalable read mapping by seed-and-vote. *Nucleic Acids Res.* **41**, e108. 10.1093/nar/gkt214 (2013). 23558742 10.1093/nar/gkt214 PMC3664803

[65] Robinson, M. D., McCarthy, D. J. & Smyth, G. K. EdgeR: a bioconductor package for differential expression analysis of digital gene expression data. *Bioinformatics* **26**, 139–140. 10.1093/bioinformatics/btp616 (2010). 19910308 10.1093/bioinformatics/btp616 PMC2796818

[66] Fang, H., Knezevic, B., Burnham, K. L. & Knight, J. C. XGR software for enhanced interpretation of genomic summary data, illustrated by application to immunological traits. *Genome Med.* **8**, 129. 10.1186/s13073-016-0384-y (2016). 27964755 10.1186/s13073-016-0384-y PMC5154134

[67] Alsaigh, T., Evans, D., Frankel, D. & Torkamani, A. Decoding the transcriptome of calcified atherosclerotic plaque at single-cell resolution. *Commun. Biol.* **5**, 1084. 10.1038/s42003-022-04056-7 (2022). 36224302 10.1038/s42003-022-04056-7 PMC9556750

[68] Langmead, B. & Salzberg, S. L. Fast gapped-read alignment with bowtie 2. *Nat. Methods*. **9**, 357–359. 10.1038/nmeth.1923 (2012). 22388286 10.1038/nmeth.1923 PMC3322381

[69] Quinlan, A. R. & Hall, I. M. BEDTools: a flexible suite of utilities for comparing genomic features. *Bioinformatics* **26**, 841–842. 10.1093/bioinformatics/btq033 (2010). 20110278 10.1093/bioinformatics/btq033 PMC2832824

[70] Zhang, Y. et al. Model-based analysis of ChIP-Seq (MACS). *Genome Biol.* **9**, R137. 10.1186/gb-2008-9-9-r137 (2008). 18798982 10.1186/gb-2008-9-9-r137 PMC2592715

[71] Lun, A. T. & Smyth, G. K. Csaw: a bioconductor package for differential binding analysis of ChIP-seq data using sliding windows. *Nucleic Acids Res.* **44**, e45. 10.1093/nar/gkv1191 (2016). 26578583 10.1093/nar/gkv1191 PMC4797262

[72] van der Auwera, G. & O’Connor, B. D. *Genomics in the Cloud: Using Docker, GATK, and WDL in Terra* (O’Reilly Media, 2020).

[73] Wang, C. et al. Integrative analyses of single-cell transcriptome and regulome using MAESTRO. *Genome Biol.* **21**, 198. 10.1186/s13059-020-02116-x (2020). 32767996 10.1186/s13059-020-02116-x PMC7412809

[74] Khan, A. et al. JASPAR 2018: update of the open-access database of transcription factor binding profiles and its web framework. *Nucleic Acids Res.* **46**, D1284. 10.1093/nar/gkx1188 (2018). 29161433 10.1093/nar/gkx1188 PMC5753202

[75] McLean, C. Y. et al. GREAT improves functional interpretation of cis-regulatory regions. *Nat. Biotechnol.* **28**, 495–501. 10.1038/nbt.1630 (2010). 20436461 10.1038/nbt.1630 PMC4840234

[76] Purcell, S. et al. PLINK: a tool set for whole-genome association and population-based linkage analyses. *Am. J. Hum. Genet.* **81**, 559–575. 10.1086/519795 (2007). 17701901 10.1086/519795 PMC1950838

[77] Consortium, G. T. The GTEx consortium atlas of genetic regulatory effects across human tissues. *Science* **369**, 1318–1330. 10.1126/science.aaz1776 (2020). 32913098 10.1126/science.aaz1776 PMC7737656

[78] Finucane, H. K. et al. Partitioning heritability by functional annotation using genome-wide association summary statistics. *Nat. Genet.* **47**, 1228–1235. 10.1038/ng.3404 (2015). 26414678 10.1038/ng.3404 PMC4626285

[79] Gazal, S. et al. Linkage disequilibrium-dependent architecture of human complex traits shows action of negative selection. *Nat. Genet.* **49**, 1421–1427. 10.1038/ng.3954 (2017). 28892061 10.1038/ng.3954 PMC6133304

