# Supplementary material for: Ox-LDL induces a non-inflammatory response enriched for coronary artery disease risk in human endothelial cells
Source: Sci Rep. 2025 Jul 1;15:21877. doi: 10.1038/s41598-025-07763-3 (PMC12219569; doi:10.1038/s41598-025-07763-3)
Supplement: Supplementary file 2 — Supplementary Material 2 [file 41598_2025_7763_MOESM2_ESM.pdf]

# Supplementary Materials

## Ox-LDL induces a non-inflammatory response enriched for coronary artery disease risk in human endothelial cells

Jiahao Jiang[1], Thomas K. Hiron[1,2], Anil Chalisey[1,3], Yashaswat Malhotra[1], Thomas Agbaedeng[1], Chris A. O'Callaghan[1]

[1] Centre for Human Genetics, Nuffield Department of Medicine, University of Oxford, Oxford, UK
[2] Present address: Department of Physiology, Anatomy & Genetics, University of Oxford, Oxford, UK

[3] Present address: Cambridge University Hospitals NHS Foundation Trust, Cambridge, UK

Correspondence to chris.ocallaghan@ndm.ox.ac.uk

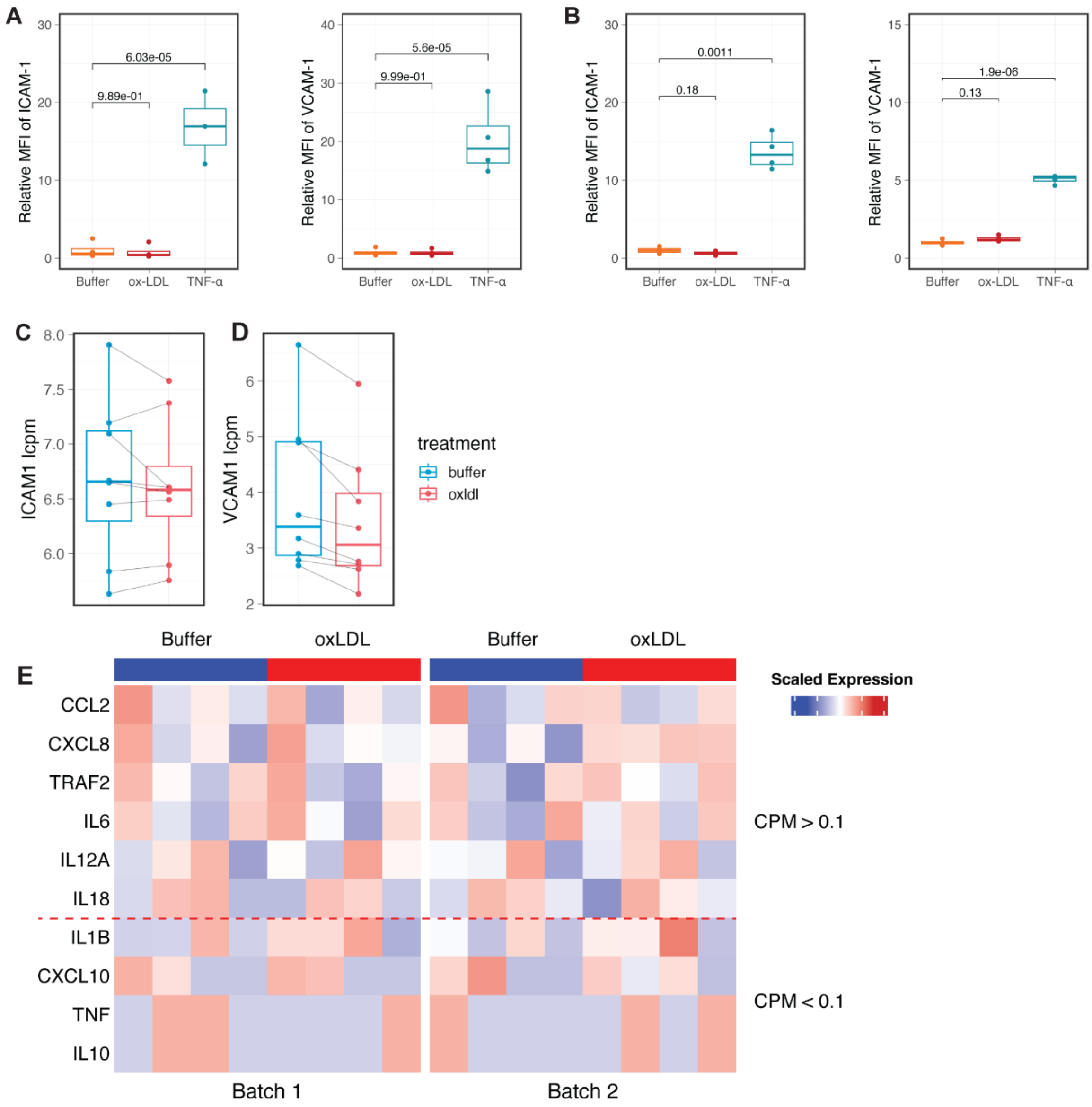


**Figure S1 Ox-LDL alone does not lead to a typical activated endothelial phenotype.** **A**, Quantification of membrane ICAM-1 (left) and VCAM-1 (right) level using flow cytometry in HAECs treated with 50ug/mL ox-LDL for 24 hours (n=4 biological replicates). HAECs treated with 10ng/mL TNF- α for 24 hours were included as a positive control. Signals were normalised to the buffer group and shown as the relative mean fluorescence intensity. P-values were calculated using one-way ANOVA with post-hoc Dunnett's Tests. **B**, same as **A** but measured with and without 48 hours of ox-LDL / TNF- α treatment. **C** and **D**, Normalised log-count-per-million gene expression of *ICAM1* (**C**) and *VCAM1* (**D**) as quantified by bulk RNA-seq, coloured by treatment group. HAECs were treated with 50ug/mL ox-LDL for 48 hours for RNA-seq experiments (n=4 biological replicates with 2 technical repeats per donor). Paired samples from the same donor are connected by lines. **E**, Heatmap showing inflammatory gene expression in buffer or ox-LDL-treated HAECs

from two independent batches. Gene expression levels are scaled across samples for visualization purposes. Genes are ordered by their average expression, including 4 genes that were not included in the differential expression analysis due to low expression (CPM, counts per million < 0.1). Of these 10 genes, only CXCL8 showed nominal significance (p=0.036), but this was not significant after adjustment for multiple testing (FDR-adjusted p = 0.274).

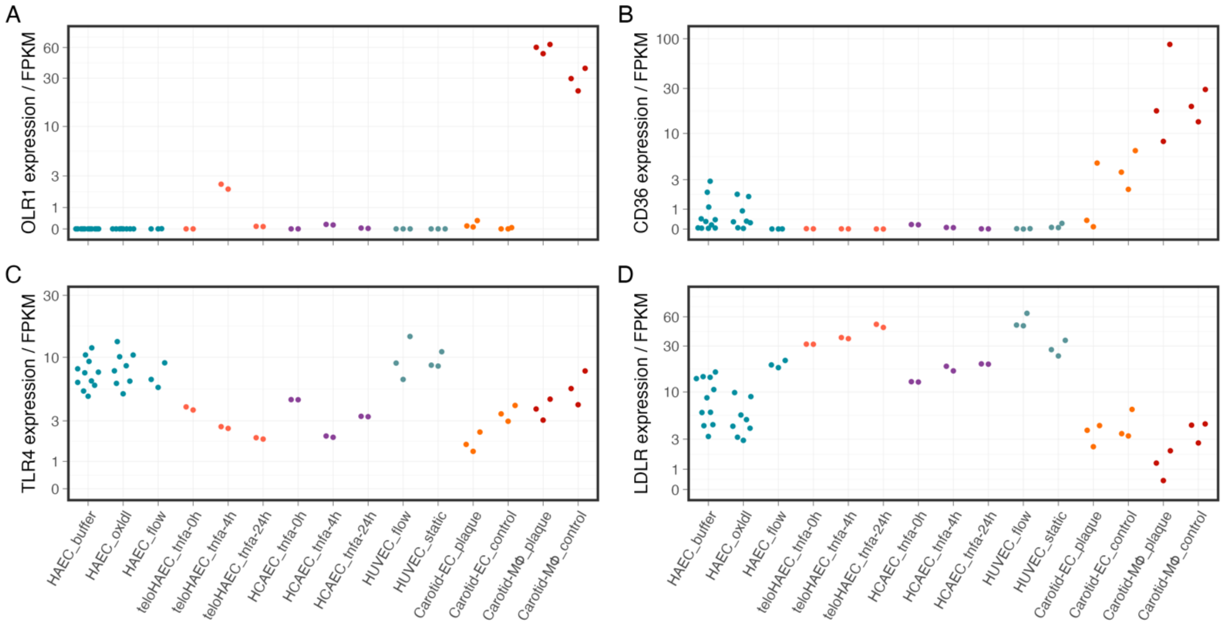


**Figure S2 *OLR1* is only weakly expressed in human endothelial cells under physiological conditions.** The mRNA levels of *OLR1* (**A**), *CD36* (**B**), *TLR4* (**C**) *and LDLR* (**D**) were examined across human endothelial cells of various origins including disease-free primary (HAEC) and immortalized aortic endothelial cell (teloHAEC) [1], coronary artery endothelial cell (HCAEC) [1], and umbilical vein endothelial cell (HUVEC) [2]. Pseudo-bulk expression profiles of *in vivo* endothelial cells and macrophages from advanced carotid atherosclerotic plaque or adjacent disease-free tissue [3] are shown to the right for comparison. All HAEC datasets were generated in this study, including unpublished data on cells grown under laminar shear stress (HAEC_flow).

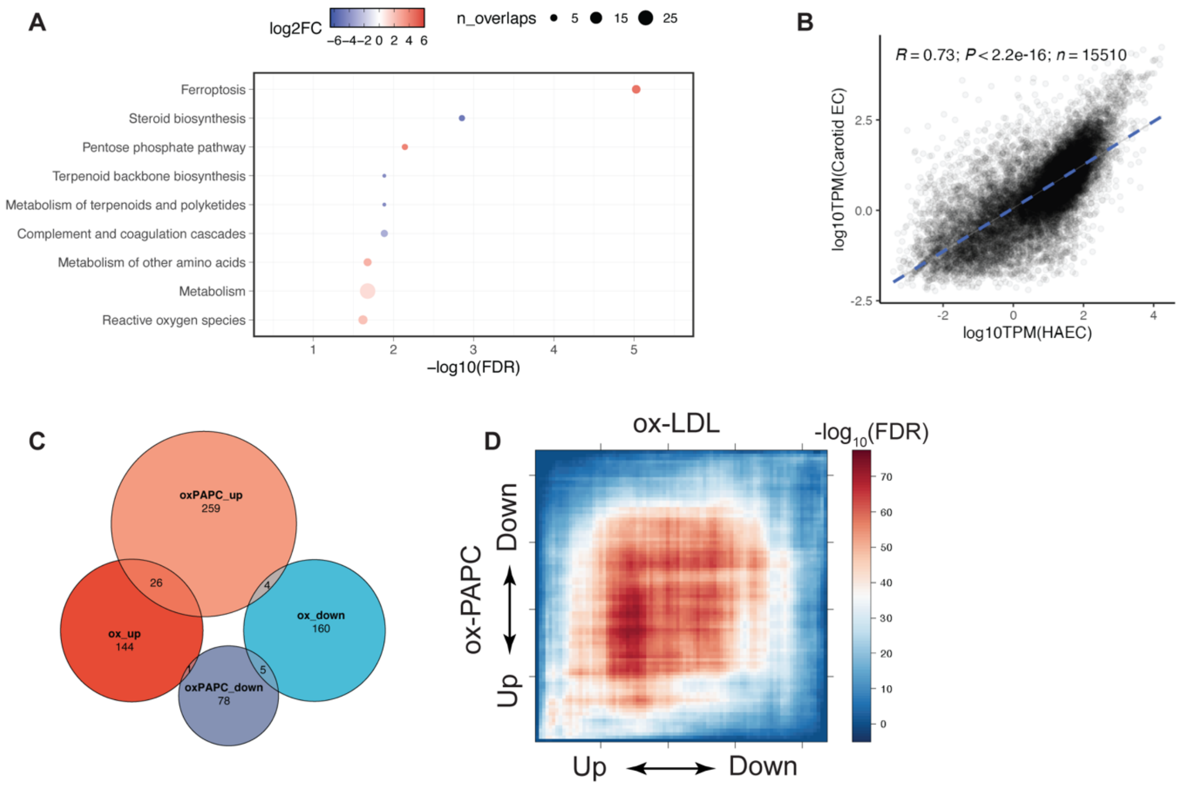


**Figure S3 Limited overlaps between the transcriptomic response to ox-LDL and ox-PAPC in HAECs. A**, KEGG pathways [4] enriched in the differentially expressed genes upon ox-LDL exposure, ranked by statistical significance. Colour represents log2 fold change of pathway enrichment and size represents the number of overlapped genes in each pathway. **B**, Scatter plot comparing gene expression profiles of human carotid artery endothelial cells *in vivo* [3] (pseudobulk scRNA-seq) versus primary human aortic endothelial cell *ex vivo* (bulk RNA-seq). 10,179 of the 11,393 genes expressed at TPM > 1 in healthy carotid EC are also expressed at TPM > 1 in HAECs. R and p-values from two-sided Pearson correlation test. **C**, Venn plot showing the overlaps between genes regulated by ox-LDL and ox-PAPC in HAECs. **D**, Rank-rank hypergeometric overlap tests showing the lack of overlaps in genes significantly regulated by ox-LDL (x axis) and ox-PAPC (y axis). The colour denotes the significance of each hypergeometric test ($-\log_{10}$FDR), measuring the extent of overlap between the two gene set on a sliding basis. Note the lack of significance at the end of x and y axes, indicating limited overlaps between differentially-expressed genes in the two datasets.

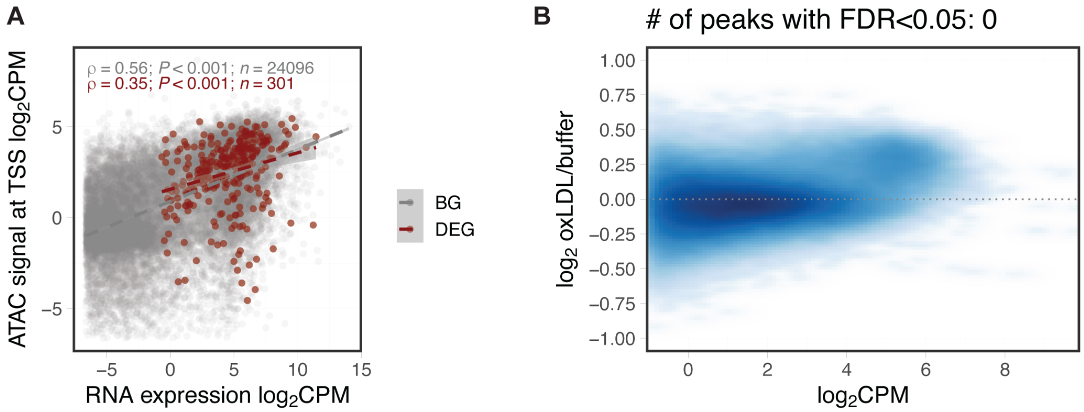


**Figure S4 Ox-LDL does not induce global changes in chromatin accessibility in HAECs. A**, Correlation between gene expression and chromatin accessibility at the 2-kb window centred at the transcription start site (TSS) in differentially expressed genes by ox-LDL (red) and all other genes (grey). **B**, Fold change in chromatin accessibility plotted against the average accessibility across all open chromatin regions in HAECs. No open chromatin peak is identified as differentially accessible under the FDR threshold of 0.05.

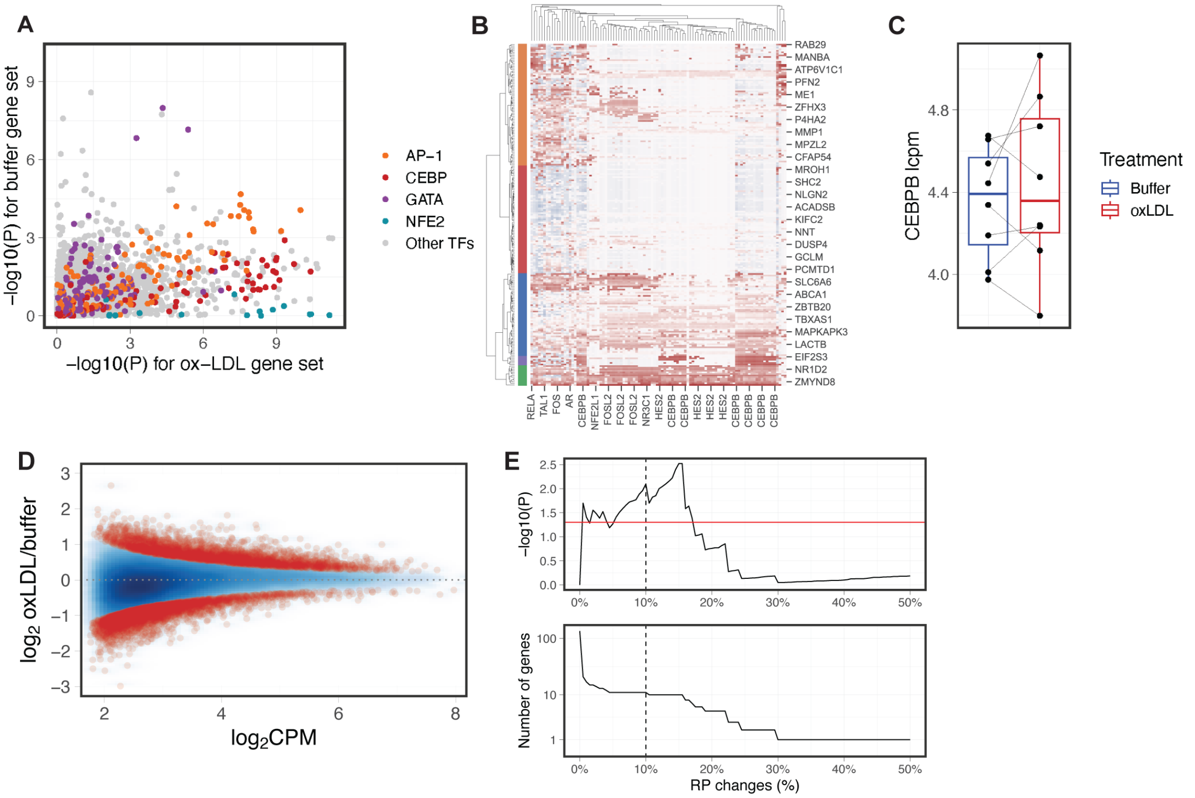


**Figure S5 Ox-LDL-specific in-silico deletion analysis highlights CEBPB as a transcription regulator in HAECs induced by ox-LDL.** A, ISD analysis was repeated using the enhancer landscape (H3K27ac ChIP-seq) instead of the open chromatin landscape (ATAC-seq), and p-values are shown similar to those in Figure 3D. **B**, Heatmap showing the predicted regulatory effect of each TF ISD (column) on the expression of individual genes (row) induced by ox-LDL treatment. The colour in the heatmap represents predicted regulatory score (in an arbitrary unit), with red denoting activating effects. **C**, Normalised log-count-per-million (lcpm) gene expression of *CEBPB* as quantified by bulk RNA-seq, coloured by treatment group. HAECs were treated with 50ug/mL ox-LDL for 48 hours for RNA-seq experiments (n=4 biological replicates with 2 technical replicates per donor). **D**, Fold change in CEBPB binding plotted against the average ChIP-seq signals across all CEBPB binding sites in primary human macrophages. A subset of 8,718 differential binding sites under the p-value threshold of 0.05 (coloured in red) were selected for further ISD analysis. **E**, Enrichment test for genes disrupted by the ox-LDL-specific ISD in ox-LDL up-regulated genes. Disrupted genes are selected based on a sliding threshold of changes in regulatory potential, and the threshold used in Figure 3E is marked by the dashed line.

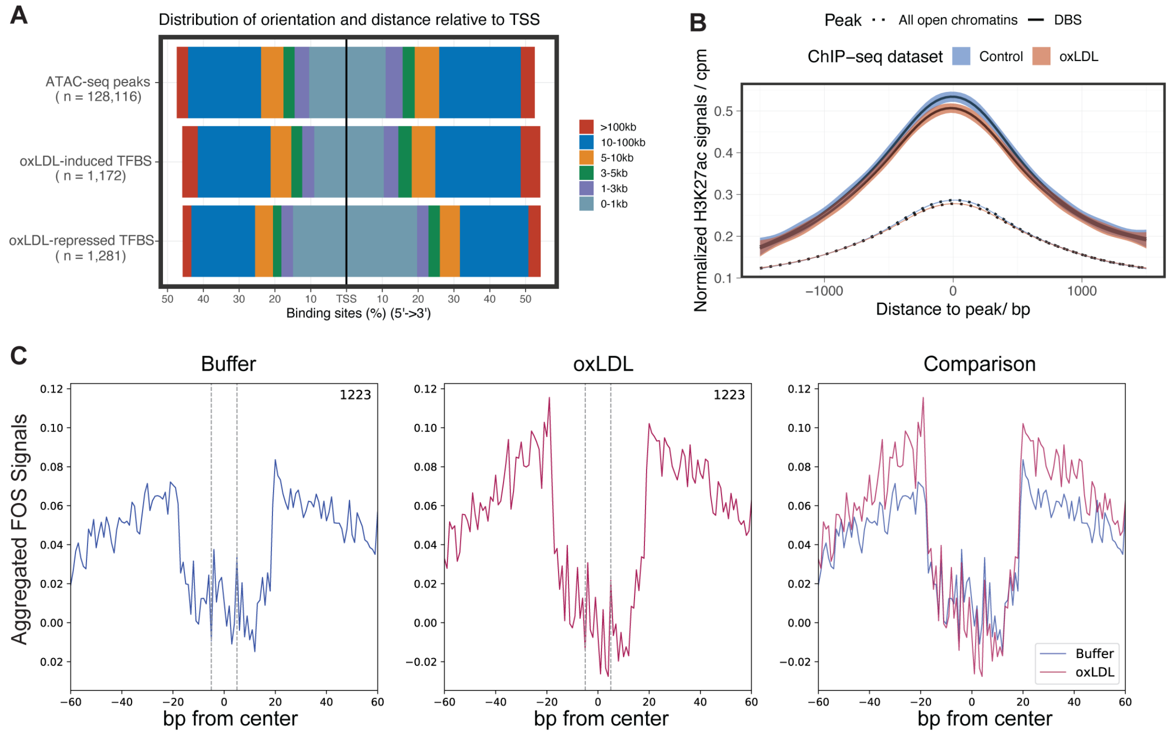


**Figure S6 Motif footprinting analysis reveals redistribution of transcription factor binding in ox-LDL-exposed endothelial cells. A**, Genomic annotation of the identified TF binding sites induced or repressed by ox-LDL. **B**, Aggregated H3K27ac signals at peaks containing dynamic TF binding sites (DBS) or background open chromatin regions. Signals from unexposed (blue) and ox-LDL-exposed (red) HAECs are plotted separately. Shaded regions indicate 95% confidence intervals. **C**, Aggregated foot print signals for FOS at n=1,223 potential binding sites with ox-LDL-induced activity.

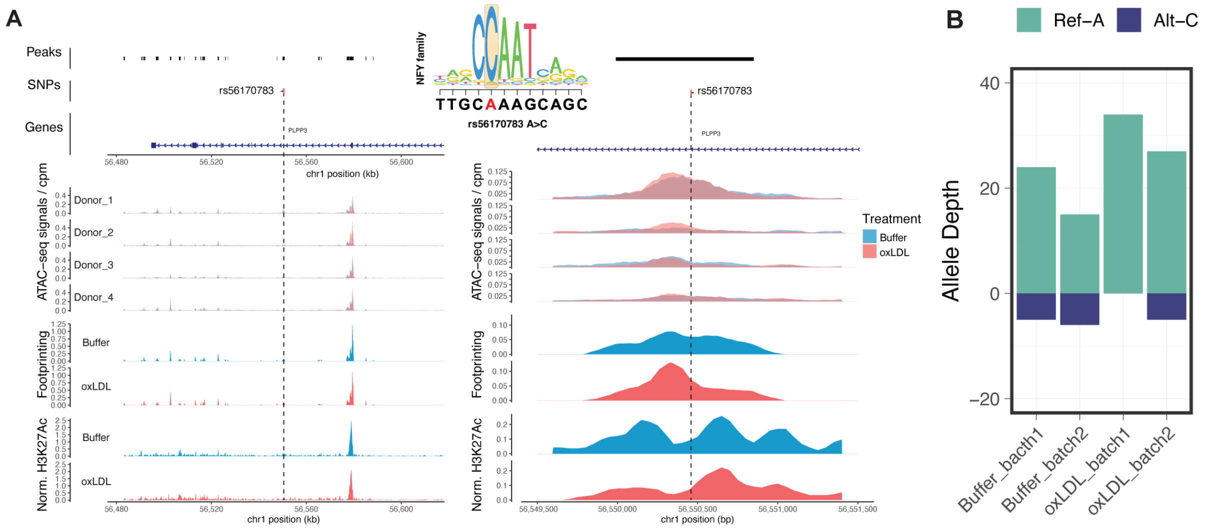


**Figure S7 The regulatory landscape at the prioritised *PLPP3* locus.**

**A**, The candidate causal variant rs56170783 is marked in red. Tracks (from top to bottom) showing: ATAC-seq peaks (peaks), CAD-associated variants (SNPs), genomic annotations (Genes), ATAC-seq signals aggregated by biological donors with signals from paired samples overlaid together, Motif footprinting score aggregated by treatment, and enhancer signals from H3K27ac ChIP-seq. Higher resolution coverage plots centred on the risk variant are plotted in the right panel. **B**, Allelic sequencing coverage at rs56170783 in the ATAC-seq libraries of four heterozygous samples.

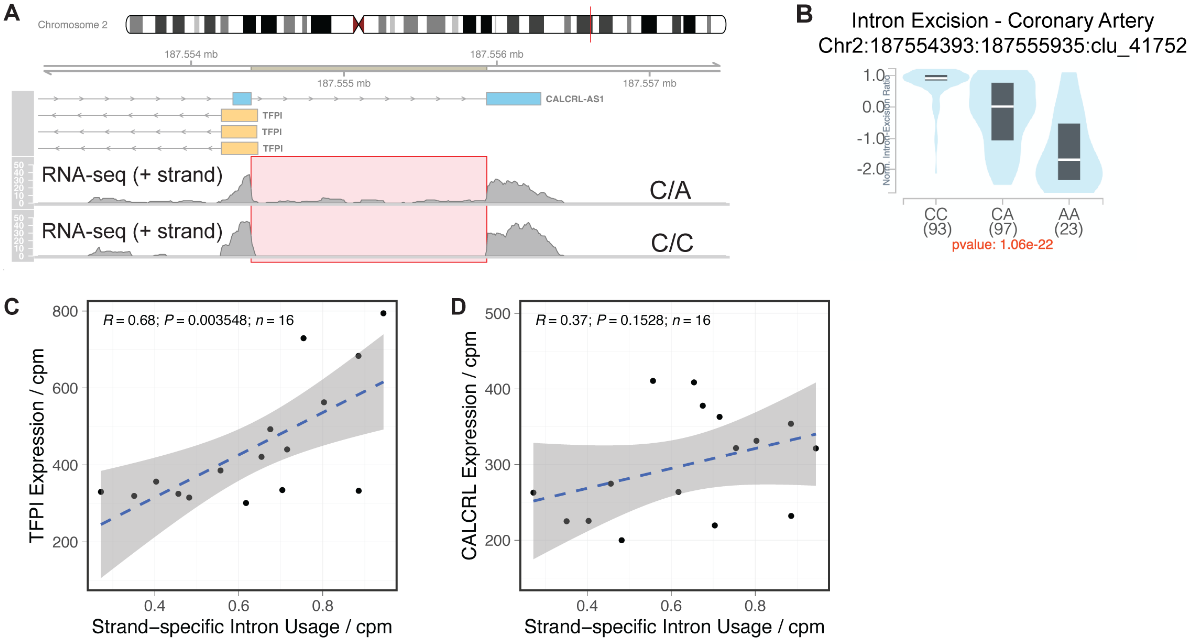


**Figure S8 Alternative splicing of CALCRL-AS1 is associated with gene expression change in TFPI but not CALCRL. A**, Overview of the alternative splicing event of the last intron of *CALCRL-AS1*. Coverage plots of RNA-seq reads on the positive strand are plotted in the bottom panel. The sample heterozygous for rs62172376 shows retention of the last intron, as highlighted in red, which is antisense to the promoter region of *TFPI*. **B**, Normalised excision ratio for the intron chr2-187554393-187555935 of *CALCRL-AS1* as shown in **A** in human coronary artery based on the GTEx data. Samples are grouped by the genotype at the SNP rs62172376. **C** and **D**, Pearson correlation tests between the strand-specific usage of the intron chr2-187554393-187555935 and gene expression of *TFPI* (**C**) and *CALCRL* (**D**). Gene and intron expression normalised to sequencing depth (count-per-million-reads). Shaded regions indicate 95% confidence intervals estimated from n=16 HAEC samples from 4 biological donors sequenced in this study.

# References

1 Lalonde, S. *et al*. Integrative analysis of vascular endothelial cell genomic features identifies AIDA as a coronary artery disease candidate gene. *Genome Biol* **20**, 133 (2019). https://doi.org:10.1186/s13059-019-1749-5

2 Buglak, D. B. *et al*. Nuclear SUN1 stabilizes endothelial cell junctions via microtubules to regulate blood vessel formation. *Elife* **12** (2023). https://doi.org:10.7554/eLife.83652

3 Alsaigh, T., Evans, D., Frankel, D. & Torkamani, A. Decoding the transcriptome of calcified atherosclerotic plaque at single-cell resolution. *Commun Biol* **5**, 1084 (2022). https://doi.org:10.1038/s42003-022-04056-7

4 Kanehisa, M., Furumichi, M., Sato, Y., Matsuura, Y. & Ishiguro-Watanabe, M. KEGG: biological systems database as a model of the real world. *Nucleic Acids Res* **53**, D672-D677 (2025). https://doi.org:10.1093/nar/gkae909
